# Context dependent variation in corticosterone and phenotypic divergence of *Rana arvalis* populations along an acidification gradient

**DOI:** 10.1186/s12862-022-01967-1

**Published:** 2022-02-05

**Authors:** Jelena Mausbach, Anssi Laurila, Katja Räsänen

**Affiliations:** 1 Department of Aquatic Ecology, Eawag, Ueberlandstrasse 133, 8600 Duebendorf, Switzerland; 2 Institute of Integrative Biology, ETH Zurich, Universitätstrasse 16, 8092 Zurich, Switzerland; 3 Animal Ecology/Department of Ecology and Genetics, Evolutionary Biology Centre, Uppsala University, Norbyvägen 18D, 75236 Uppsala, Sweden; 4 Department of Biological and Environmental Science, University of Jyväskylä, Survontie 9C, 40014 Jyväskylä, Finland

**Keywords:** Acidification, Adaptive divergence, Amphibians, Corticosterone, Environmental stress, Evolutionary physiology, Phenotypic plasticity

## Abstract

**Background:**

Physiological processes, as immediate responses to the environment, are important mechanisms of phenotypic plasticity and can influence evolution at ecological time scales. In stressful environments, physiological stress responses of individuals are initiated and integrated via the release of hormones, such as corticosterone (CORT). In vertebrates, CORT influences energy metabolism and resource allocation to multiple fitness traits (e.g. growth and morphology) and can be an important mediator of rapid adaptation to environmental stress, such as acidification. The moor frog, *Rana arvalis,* shows adaptive divergence in larval life-histories and predator defense traits along an acidification gradient in Sweden. Here we take a first step to understanding the role of CORT in this adaptive divergence. We conducted a fully factorial laboratory experiment and reared tadpoles from three populations (one acidic, one neutral and one intermediate pH origin) in two pH treatments (Acid versus Neutral pH) from hatching to metamorphosis. We tested how the populations differ in tadpole CORT profiles and how CORT is associated with tadpole life-history and morphological traits.

**Results:**

We found clear differences among the populations in CORT profiles across different developmental stages, but only weak effects of pH treatment on CORT. Tadpoles from the acid origin population had, on average, lower CORT levels than tadpoles from the neutral origin population, and the intermediate pH origin population had intermediate CORT levels. Overall, tadpoles with higher CORT levels developed faster and had shorter and shallower tails, as well as shallower tail muscles.

**Conclusions:**

Our common garden results indicate among population divergence in CORT levels, likely reflecting acidification mediated divergent selection on tadpole physiology, concomitant to selection on larval life-histories and morphology. However, CORT levels were highly environmental context dependent. Jointly these results indicate a potential role for CORT as a mediator of multi-trait divergence along environmental stress gradients in natural populations. At the same time, the population level differences and high context dependency in CORT levels suggest that snapshot assessment of CORT in nature may not be reliable bioindicators of stress.

**Supplementary Information:**

The online version contains supplementary material available at 10.1186/s12862-022-01967-1.

## Background

Environmental change, be it natural or anthropogenic, is often associated with the exposure of individuals and populations to abiotic and biotic environmental stressors, which can lead to strong natural selection [1]. At short evolutionary time scales, environmental stress can lead to “rapid evolution” [2], raising the questions how natural selection acts on multiple interacting traits and what are the mechanisms of rapid adaptation? [1] In order to understand eco-evolutionary responses of populations to stressful and fast changing environments, we need to understand how environmental and genetic effects jointly act on the organismal phenotype [1, 3–5].

A major source of environmental responsiveness of organisms is physiological plasticity, which determines the immediate responses and the ability of individuals to acclimate to environmental stress [6–10]. Physiological responses are, hence, expected to be under natural selection [8, 11–13], whereby genotypes with optimal combinations of stress responses and energy metabolism for a given ecological context should be favoured [14, 15]. Although the role of physiology in adaptation has received attention in ecophysiology [16] and evolutionary physiology [17, 18], it has yet to be fully integrated across fields (i.e. as eco-evolutionary physiology of contemporary populations).

In vertebrates, a candidate pathway in integrated stress responses arises via the glucocorticoid hormones corticosterone (CORT) and/or cortisol [19]. In addition to stress responses, these glucocorticoids are involved in general metabolic processes and a range of gene expression networks (metabolism, growth, tissue repair, reproduction, immune function; reviewed in [19, 20]), and can therefore be under strong natural selection. Consequently, glucocorticoids are major mediators of the phenotype and of special interest in the context of eco-evolutionary physiology [3, 19, 20].

CORT is secreted after the activation of the hypothalamic–pituitary–adrenal (HPA) axis which, among other functions, is one main physiological pathway responsible for stress responses in vertebrates [19, 20]. In the short-term, elevated glucocorticoid levels can allow energy mobilization in stressful situations (e.g. via fat catabolism and decrease in digestion; [19]). However, chronically elevated CORT levels can be costly and cause reproductive malfunction, cellular damage and immunosuppression [19, 21–25]. Therefore, under long-term exposure to stress, populations face a trade-off: natural selection should prevent detrimental effects of elevated CORT levels, yet maintain the ability to respond adaptively to temporally varying stressors (e.g. predation attempts or extreme temperatures). In general, natural selection on CORT levels may act on both “supportive” (i.e. maintaining ability to respond by elevating CORT levels) and “protective” (i.e. reducing negative effects of elevated CORT) processes [14]. Thereby selection may favour either higher (supportive process) or lower (protective process) CORT levels in energetically challenging or stressful environments, and act on baseline and/or stress induced CORT levels in a context dependent manner (i.e. depending on costs versus benefits; [14]).

In amphibians, environmental stress activates the hypothalamic-pituitary-interrenal (HPI) axis, leading to the secretion of CORT [26–29]. In tadpoles, CORT levels are influenced by many different stressors, such as acidity, predators and parasites [30–32], and CORT can influence many fitness traits, from growth rates and immune function to traits related to resource acquisition and predator defense [31]. CORT levels can also vary strongly across the developmental stages, generally peaking at metamorphosis [33]. At early to mid-larval stages elevated CORT levels may decrease growth and development rates [34–36], as well as reduce body length and increase tail depth [30, 31]. From late to mid-larval stages elevated CORT levels may instead accelerate development [26, 37]. CORT related performance trade-offs [31, 35, 36] and geographic variation in glucocorticoid levels has been demonstrated [14, 30, 34], indicating the potential for divergent natural selection through CORT. However, how CORT profiles and CORT—trait associations vary across divergent environments in natural populations is poorly known.

Environmental acidity, both natural and anthropogenic, is stressful for a range of organisms [38, 39], including amphibians [reviewed in 40], and can be a potent agent of natural selection. Moor frog, *Rana arvalis,* populations along a pH gradient in Sweden show phenotypic divergence in multiple tadpole traits [41, 42]. Specifically, laboratory studies show that under common garden conditions, tadpoles from acid origin populations develop slower, grow faster and are larger at metamorphosis, and have deeper tails, than tadpoles from neutral origin populations [41, 43]. This divergence is mediated through a combination of maternal and direct genetic effects [43, 44] and is a response to both acidity and predator induced divergent selection [41]. However, the physiological underpinnings of this multi-trait divergence are unknown.

Here we aim to increase the understanding of the role of glucocorticoids in adaptive divergence of natural populations. Specifically, we study the effects of acid stress on CORT levels, and associations between CORT and functionally relevant traits (life-history and morphology), in *R. arvalis* tadpoles from three divergent populations. In a fully factorial laboratory experiment, we reared tadpoles from an acidic (Tottatjärn, TT), a neutral (Rud, RD) and an intermediate (Bergsjön, BS) pH origin population in two contrasting pH treatments: Acid pH (physiologically stressful) and Neutral pH (physiologically benign). We compared tadpoles from these population-treatment combinations at three developmental stages (mid-larval stages G32 and G38, and metamorphosis, G42, [45]). We made the following predictions. First, if acidic pH is stressful and CORT is an indicator of stress, tadpoles in the Acid pH treatment should show *elevated* CORT levels relative to the Neutral pH treatment. Second, if there has been divergent selection on either baseline (e.g. due to differential metabolic demands) or stress induced CORT [14], tadpoles from the acidic and neutral pH origin population should differ in their CORT profiles. This phenotypic divergence among the populations could be in the form of Genotype × Environment interactions (i.e. seen as differential CORT responses of populations to the pH treatments) and/or differences in mean CORT levels (independent of pH treatment). Finally, if CORT is a key mediator of multi-trait adaptive divergence, CORT levels should correlate with larval life-history traits and tail morphology (latter representing a typical inducible predator defence trait, [41]). In particular, we expected elevated CORT levels to correlate with larval developmental time, body size and tail morphology.

## Results

**Fig. 1:**
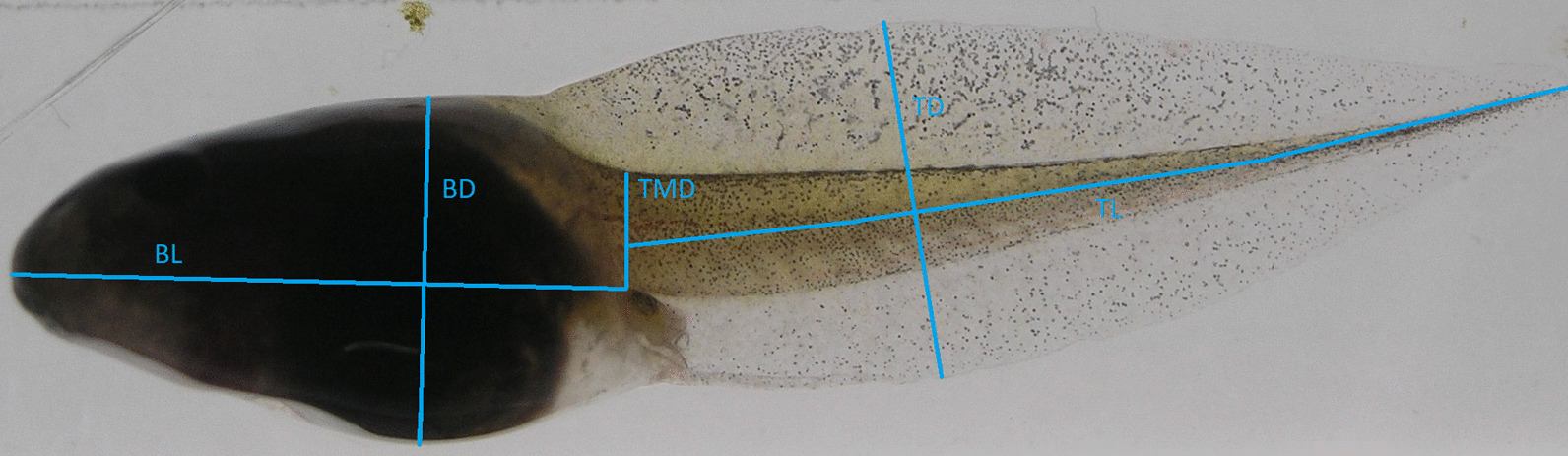
Morphological traits of *Rana arvalis* tadpoles measured at G32 and G38: body length (BL), body depth (BD), tail length (TL), maximum tail depth (TD) and tail muscle depth (TMD). BL was taken from mouth to the base of the hind leg, BD was taken where it was longest orthogonal to BL, TL was taken from base of the hind leg to tail tip, TD was measured where it is deepest and TMD was taken orthogonal to the “spine” right at its base

Tadpoles from the three phenotypically divergent populations (TT: Acid origin, RD: Neutral origin, and BS: Intermediate origin) were reared in either Acid (target pH 4.3) or Neutral (target pH 7.5) pH in the lab. We tested i) how tadpoles from the three populations differ in CORT profiles and ii) what are the CORT—trait relationships in multivariate space for larval development time (days from G25 to a given tadpole stage), tadpole size (mass, g) and tadpole morphology (Fig. 1). With regard to the core hypotheses for CORT, Population main effects would be indicative of genetic divergence in response to selection on baseline CORT levels (involved in organismal metabolism in absence of stress) or chronic stress induced CORT levels [14], pH main effects and higher CORT in the Acid pH treatment would be indicative of stress induced CORT elevation, and Population x pH interaction effects would indicate among population differences in chronic CORT stress responses. Notably, given chronic exposure (weeks to months) of tadpoles, no difference between the benign (Neutral) and stressful (Acid) pH treatment may indicate that CORT levels have returned to baseline levels (e.g. to reduce detrimental effects of chronically elevated CORT) (see Discussion).

Sampling and rearing were conducted in two Blocks (A: morning sampling/warmer temperature; B: afternoon sampling/cooler temperature) and measurements were taken at three larval stages: G32, G38 and G42 (see “Methods” for details). The intended number of replicates for each population—treatment combination was eight individuals, but the following population treatment combinations at G42 had N = 7 (TT4B and TT7B) and N = 9 (BS7B) (as detailed in Methods). Therefore, a total of 287 individuals were included in the statistical analyses. The data was analysed using univariate and multivariate AN(C)OVAs and interpreted based on differences in LS means (univariate analyses) and Hypothesis-Error (HE) plots (MANOVAs) where relevant (see Methods for details). Only final models are presented here.

### Corticosterone

**Fig. 2:**
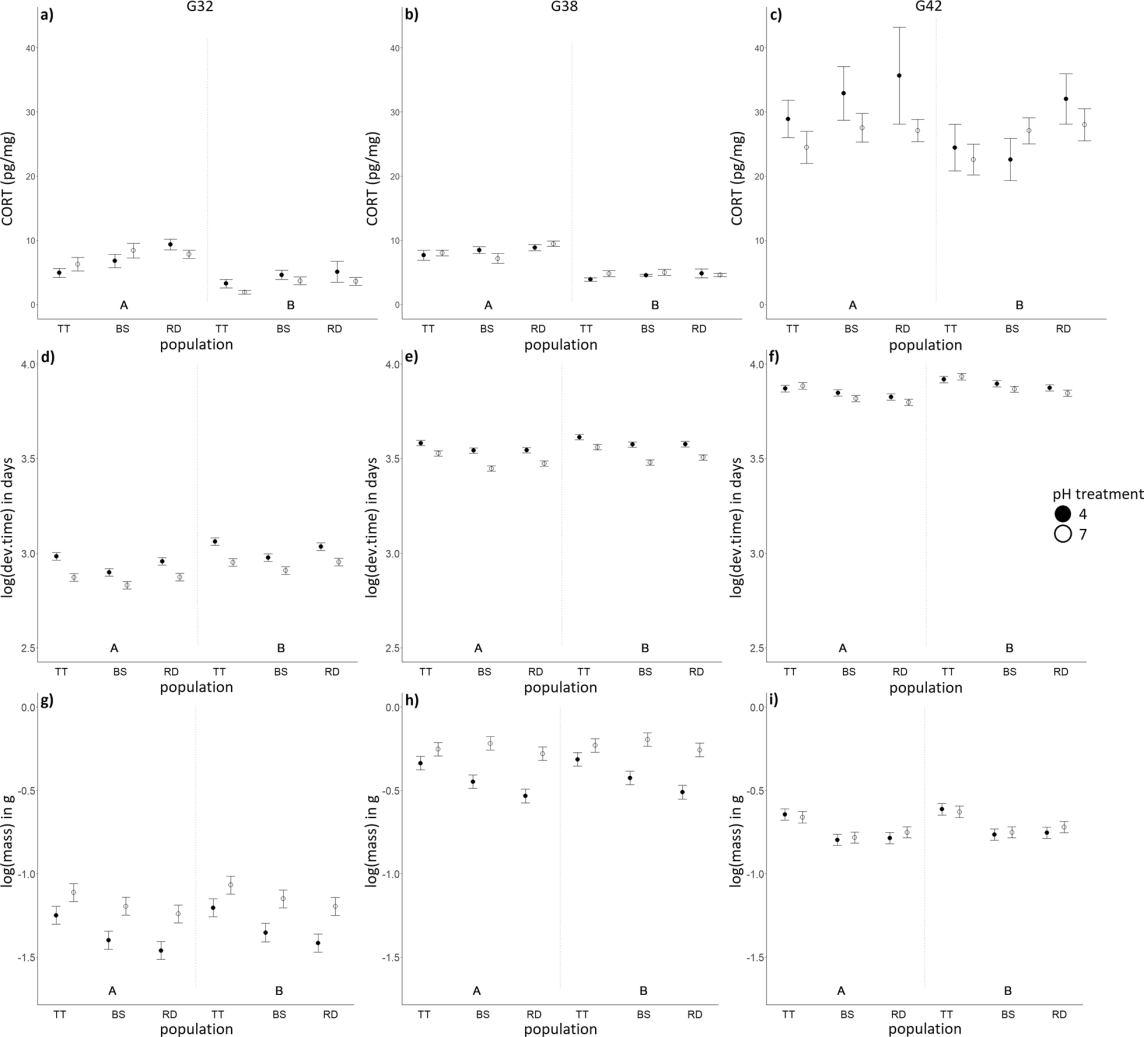
Mean ± SE of CORT levels (**a**–**c**), and LS means ± SE of log(developmental time, days from G25) (**d**–**f**) and log (mass in g) (**g**–**i**), across three larval developmental stages (G32, left panel, G38, middle panel, and G42, right panel) in *Rana arvalis*. Tadpoles from the Acid (TT), Neutral (RD) and Intermediate (BS) origin population were reared in Acid (4) or Neutral (7) pH treatment across two rearing Blocks (A: morning sampling/warmer; B: afternoon sampling/colder). Sample size was N = 8 except for the following cases: **a**) TT4B, TT7A and TT7B: N = 7; **b**) RD4B and BS4B: N = 6, BS4A and TT4B: N = 7; **c**) BS4B and TT4B: N = 6, TT7B: N = 7; **f**) TT4B and TT7B: N = 7, BS7B: N = 9; **g**) BS4B: N = 7; **i**) TT4B and TT7B: N = 7, BS7B: N = 9 (see Methods for details)

G32 and G38 tadpoles had, on average, lower CORT levels than the G42 metamorphs (Fig. 2a–c, Additional file 1: Table 1.1A). However, univariate analyses of log(CORT) across developmental stages revealed a significant pH treatment × Block × Stage interaction (Additional file 1: Table 1.1A)—indicating that CORT variation was context dependent. To examine this three-way interaction further, we next conducted models separately within each of the developmental stages (G32, G38 and G42).

At G32, there were significant Population, Block and pH treatment × Block effects (Table 1): Acid origin (TT) tadpoles had, on average, lower CORT levels than Neutral (RD) and Intermediate (BS) origin tadpoles (Tukey test, Table 1, Fig. 2a). Moreover, CORT levels were higher in the Acid than the Neutral pH treatment in the B block, whereas there was no difference between the pH treatments in the A block (Fig. 2a, Tukey test Table 1). At G38, only the Block effect was significant, with tadpoles in the A block having higher CORT levels than tadpoles in the B block (Fig. 2b, Table 1). At G42 no statistically significant effects on CORT were found (Fig. 2c, Table 1).

**Table 1:** Univariate models of log(CORT) within G32, G38 and G42 stages

Factors	Developmental stage								
	*G32*			*G38*			*G42*		
	*df*	*F*	*p*	*df*	*F*	*p*	*df*	*F*	*p*
Population	2	5.71	**0.005**	2	2.60	0.081	2	1.55	0.218
pH treatment	1	0.02	0.880	1	0.06	0.800	1	1.45	0.233
Block	1	15.15	** < 0.001**	1	152.51	** < 0.001**	1	2.10	0.151
Population × pH	2	0.34	0.712	2	1.36	0.262	2	0.52	0.596
pH × Block	1	4.84	**0.031**	–	–	–	–	–	–
Residual SE and (*df*)	0.46 (85)			0.22 (83)			0.32 (84)		
Posthoc (Tukey)	LS mean ± SE Population: TT 1.21 ± 0.09 b BS 1.63 ± 0.08 a RD 1.72 ± 0.08 a Block: A 1.89 ± 0.07 a B 1.15 ± 0.07 b pH x Block: 4A 1.83 ± 0.09 a 7A 1.95 ± 0.10 a 4B 1.30 ± 0.10 b 7B 1.00 ± 0.10 b	LS mean ± SE Block: A 2.09 ± 0.03 a B 1.50 ± 0.03 b							

Results of univariate linear models on log(CORT) for G32, G38 and G42, respectively. Results are presented for *Rana arvalis* tadpoles from the Acid (TT), Neutral (RD) and the Intermediate (BS) origin population, reared in Acid (4) or Neutral (7) pH treatment and two Blocks (A: morning/warmer, B: afternoon/colder). These are final models following removal of non-significant three- or two-way interactions. Statistically significant effects (p < 0.05) are shown in bold. Different letters in posthoc tests denote significantly different LS means (Tukey tests), indicating that 4A and 4B, and 7A and 7B, differ from each other (i.e. letters do not overlap), whereas there are no differences between the pH treatments within each block

## The multivariate phenotype

### CORT and life history traits

#### Mid-larval stage G32

*MANOVAs*—Population, pH treatment and Block had significant main effects, but no significant interactive effects, on the joint variation between CORT, developmental time and tadpole mass at G32 (Additional file 1: Table 1.3). Block explained most of the variation in this multivariate space (eta^2^: 54%), followed by pH treatment (21%) and Population (18%) (Additional file 1: Table 1.3; for canonical HE analyses see Additional file 2: Table 2.1 and Fig. 2.1A). On average, TT tadpoles developed slower and were larger than RD tadpoles, with BS tadpoles being intermediate—though life-history trait variation was pH treatment and Stage dependent (Fig. 2d and g; for Univariate ANOVAs see Additional file 1: Table 1.4).

**Fig. 3:**
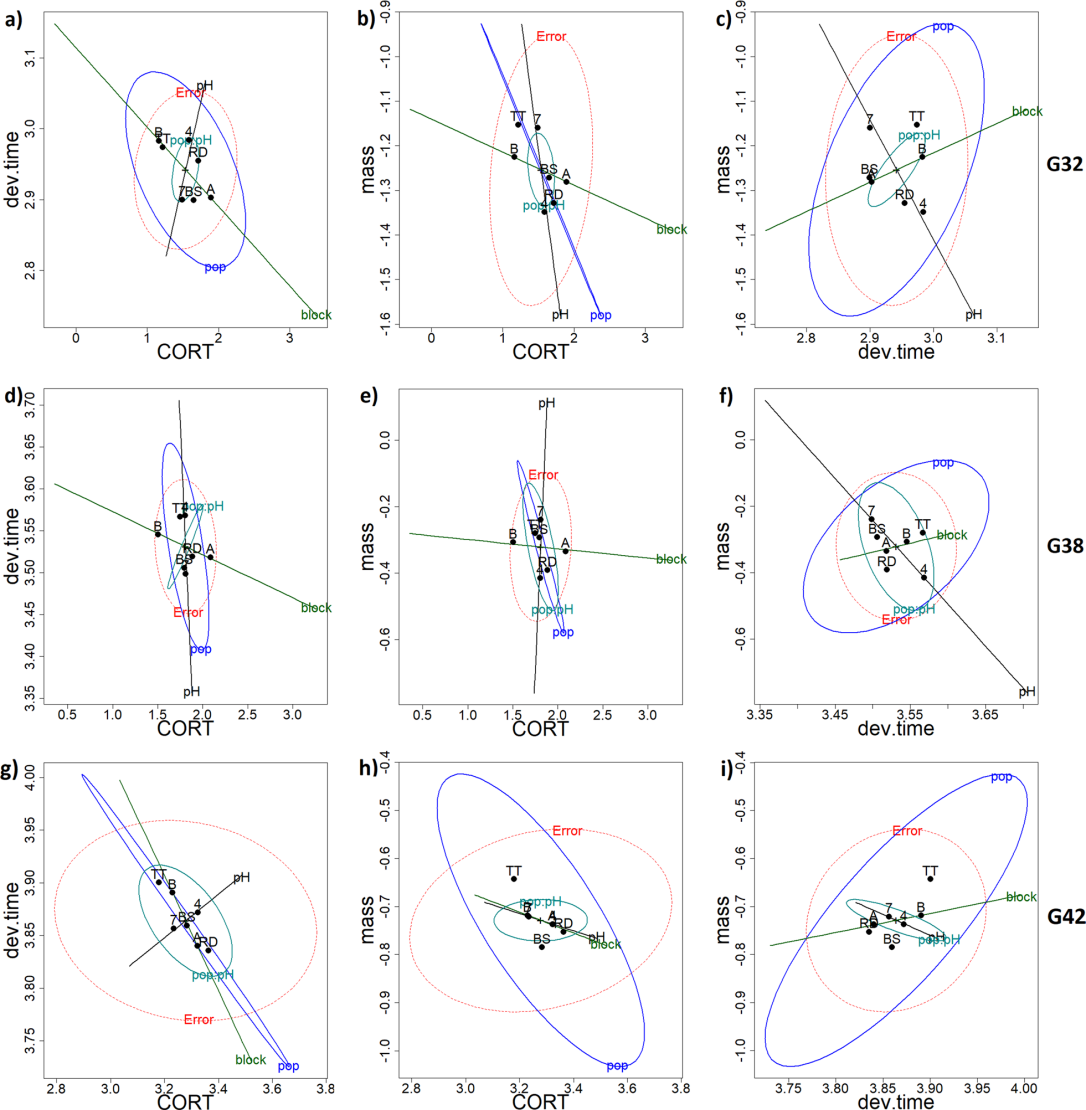
HE plots from the CORT and life history MANOVAs at larval stage G32 (**a**–**c** upper panel), G38 (**d**–**f** middle panel) and G42 (**g**–**i** lower panel) in *Rana arvalis*. All response variables were log transformed. Hypothesis ellipses that are outside of the Error ellipse (in red) indicate significant effects. The ellipses depicted are pop = Population, ph = pH treatment, pop:ph = Population-pH treatment interaction, block = rearing block. The solid dots indicate fixed effect means for Population (TT: Acid origin, RD: Neutral origin, BS: Intermediate origin), pH treatment (4: Acid, 7: Neutral) and Block (**A** morning sampling/warmer, **B** afternoon sampling/colder)

*HE-plots from the MANOVAs*—At G32, there was a strong negative association between CORT and developmental time across Blocks (Fig. 3a, block ellipsoid): individuals with higher CORT levels (A block) developed faster than those with lower CORT levels (B block). There was also a negative association between CORT and developmental time across populations (Fig. 3a, pop ellipsoid): TT individuals had lower CORT levels and developed slower, whereas RD and BS individuals had higher CORT levels and developed faster. The HE plots further indicated that the pH treatment effect in the MANOVA (Additional file 1: Table 1.3) was primarily due to tadpoles developing slower in the Acid (4) than the Neutral (7) treatment (Fig. 3c and f, pH ellipsoid), but there was no relationship between CORT and developmental time across pH treatments (Fig. 3a).

There was a subtle negative association between CORT and body mass across the Blocks (Fig. 3b, block ellipsoid): tadpoles from the A block tended to have higher CORT levels but be smaller than those from the B block. There was a stronger negative association between CORT and tadpole mass across populations: individuals with lower CORT levels were larger (TT population) than individuals with higher CORT levels (RD and BS tadpoles) (Fig. 3b, pop ellipsoid). There was no clear association between CORT and tadpole size in relation to the pH treatments.

Finally, there was a subtle positive relationship between developmental time and mass of tadpoles across Blocks (Fig. 3c): tadpoles in the A block developed faster (fewer days from G25 to G32) but were smaller, whilst tadpoles in the B block developed slower and were larger. In contrast, there was a strong positive association between developmental time and mass of tadpoles across Populations (Fig. 3c, pop ellipsoid): TT tadpoles developed slower and were larger whereas RD and BS tadpoles developed faster and were smaller. Interestingly, the pH treatment (Fig. 3c, pH ellipsoid) reversed this development time-mass relationship with individuals that developed slower (i.e. Acid, 4, treatment) being smaller than those that developed faster (i.e. Neutral, 7, treatment)—reflecting stressful conditions in the acid treatment.

#### Mid-larval stage G38

*MANOVAs*—At G38, Block, Population and pH treatment had strong and significant main effects and a significant Population x pH interaction on joint variation of CORT, developmental time and mass (Additional file 1: Table 1.3). Block explained most of the variation in this multivariate space (eta^2^: 66–67%), followed by pH treatment (46%), Population (17–18%) and Population x pH treatment (7%) (Additional file 1: Table 1.3).

*HE plots from the MANOVAs—*There was a strong negative association between CORT and developmental time across Blocks at G38 (Fig. 3d): individuals with high CORT levels (A block) developed faster than those with lower CORT levels (B block). Likewise, there was a negative association between CORT and body mass across the Blocks (Fig. 3e, block ellipsoid): tadpoles from the A block had higher CORT levels and were smaller, whereas tadpoles from the B block fell to the opposite end of the axis.

At G38, CORT was correlated with developmental time across Blocks (Fig. 3d, block ellipsoid): tadpoles that had higher CORT levels (A block) developed faster than those that had lower levels (B block). There was a strong negative relationship between developmental time and mass of tadpoles in relation to pH (Fig. 3f, pH ellipsoid): tadpoles developed slower but were smaller in the Acid pH treatment and developed faster and were larger in the Neutral pH treatment. Jointly with univariate analyses (Additional file 1: Table 1.4), the HE plots (Fig. 3a–f, and below) indicated that the pH and population effects were primarily driven by effects on development time and mass. There was a significant association between development time and mass across populations (Fig. 3f, pop ellipsoid), with TT tadpoles developing slower and being larger than RD tadpoles.

#### Metamorphosis G42

*MANOVA*—At G42, only Population and Block had a significant main effect on the joint variation of CORT, developmental time and mass (Additional file 1: Table 1.3). Variance partitioning showed that Population explained 18–20% and Block 17% of this variation (Additional file 1: Table 1.3). TT metamorphs were substantially larger in both pH treatments than RD and BS metamorphs and individuals reached metamorphosis somewhat slower in the B block (Fig. 2f and i; for univariate ANOVAs see Additional file 1: Table 1.4).

*HE plots from the MANOVAs* As for G32 and G38*,* there was a negative association between CORT and developmental time at G42 across the populations and Blocks. The Population ellipsoid (Fig. 3g and h) indicated that individuals with lower CORT levels (mostly TT tadpoles) were larger and developed slower than those with higher CORT levels (mostly BS and RD tadpoles). Individuals with higher CORT levels (i.e. A block) developed faster to metamorphosis than those with lower CORT levels (B block) (Fig. 3g, block ellipsoid).

There was a strong positive relationship between developmental time and mass across Populations (Fig. 3i, pop ellipsoid): TT metamorphs developed slower and were larger whereas RD and BS metamorphs developed faster and were smaller. There was a weak positive association between developmental time and mass at G42 across Blocks (Fig. 3i, block ellipsoid), with tadpoles that developed faster (A block) being smaller and tadpoles that developed slower being larger (B block).

The patterns of developmental time and body mass trait means seen in the HE plots (Fig. 3) for G32, G38 and G42 were confirmed in univariate models (Fig. 2, Additional file 1: Table 1.1, Table 1.4).

### CORT: morphology relationships

*Discriminant analyses of principal components (DAPC)—*A multivariate DAPC including CORT and log transformed morphological traits (body length, body depth, tail length, tail depth and tail muscle depth) across G32 and G38 showed a clear phenotypic separation of the two developmental stages (Additional file 3: Fig. 3.1, 3.2A, B & Table 3.1). As G32 is more reflective of functionally relevant mid-larval stage morphology and showed more variance across individuals in our data set (see Additional file 3), we next conducted a separate DAPC on G32 tadpoles only.

#### CORT: morphology relationships at G32

**Fig. 4:**
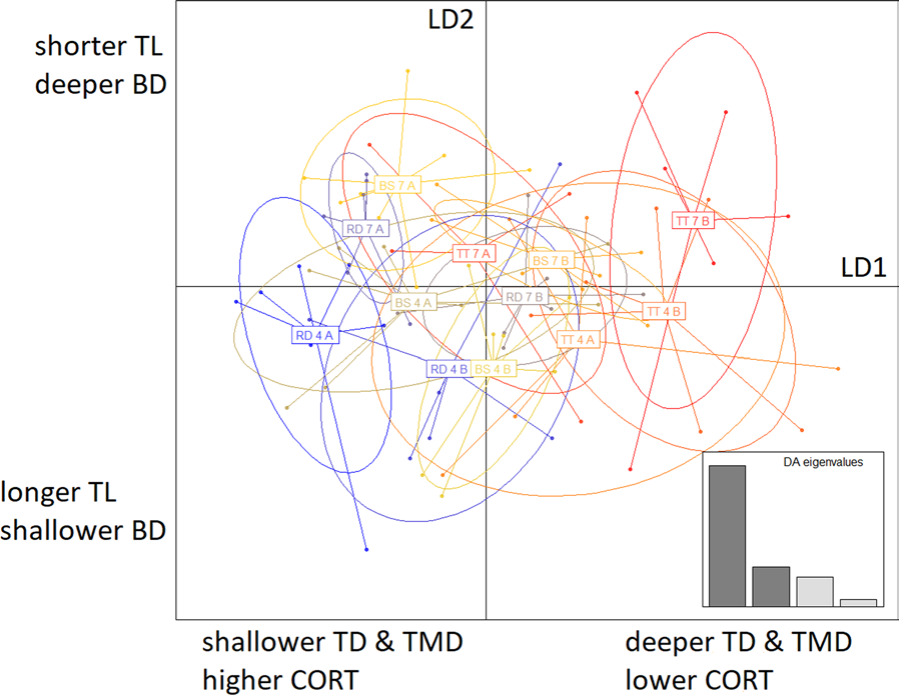
DAPC on stage G32 tadpoles for CORT and morphological traits (body length, body depth, tail length, tail depth, tail muscle depth) in *Rana arvalis*. Tadpoles from an Acid origin (TT), Intermediate origin (BS) and a Neutral origin (RD) population were reared in an Acid (here: 4) and a Neutral (here: 7) pH treatment and two Blocks (A and B). All response variables were log transformed for this analysis. LD1 represented mostly variation in tail depth (TD), tail muscle depth (TMD) and CORT, LD2 represents mainly tail length (TL) and body depth (BD) (See main text and Additional file 3 for details)

In the DAPC for G32 tadpoles, LD1 explained 64.8% of the variance, with tail muscle depth (64.6%), tail depth (18.4%) and CORT (12.2%) loading strongest on this axis. LD2 explained 18.2% of the variance, with tail length (37.9%) and body depth (29.3%) loading strongest (Fig. 4, Additional file 3: Fig. 3.1, Fig. 3.2C & Table 3.1). Visual inspection indicated that LD1 reflects mostly population level variation, with TT tadpoles having lower CORT and relatively deeper tail muscles and tails, RD tadpoles having higher CORT and relatively shallower tail muscles and tails, and BS tadpoles being intermediate (Fig. 4). LD2, on the other hand, reflected mostly variation related to the pH treatments, with tadpoles in the Acid treatment having a shallower body and longer tail and tadpoles in the Neutral treatment having a relatively deeper body and longer tail (Fig. 4).

*MANOVAs*—To investigate the multivariate relationships between CORT and tadpole morphology at G32, we next conducted a MAN(C)OVA with CORT and body length, body depth, tail length, tail muscle depth and tail depth as response variables, and mass as a covariate. All traits were log transformed. This analysis found significant Population, pH treatment, Block and mass main effects, but no significant Population x pH interaction effect (Additional file 1: Table 1.3). Partitioning of variance indicated that strongest effects on the multivariate phenotype were by mass (94%), Block (45%), Population (28–31%) and pH treatment (21%) (Additional file 1: Table 1.3) (This ranking held for Pillai’s, Wilk’s Lambda as well as Hotelling Lawley’s test statistics. Details of the canonical analyses and HEplots can be found in Additional file 2. For LSmeans of individual morphological traits, and univariate ANOVAs see Additional file 1: Fig. 1.1 and Additional file 1: Table 1.4, respectively).

**Fig. 5:**
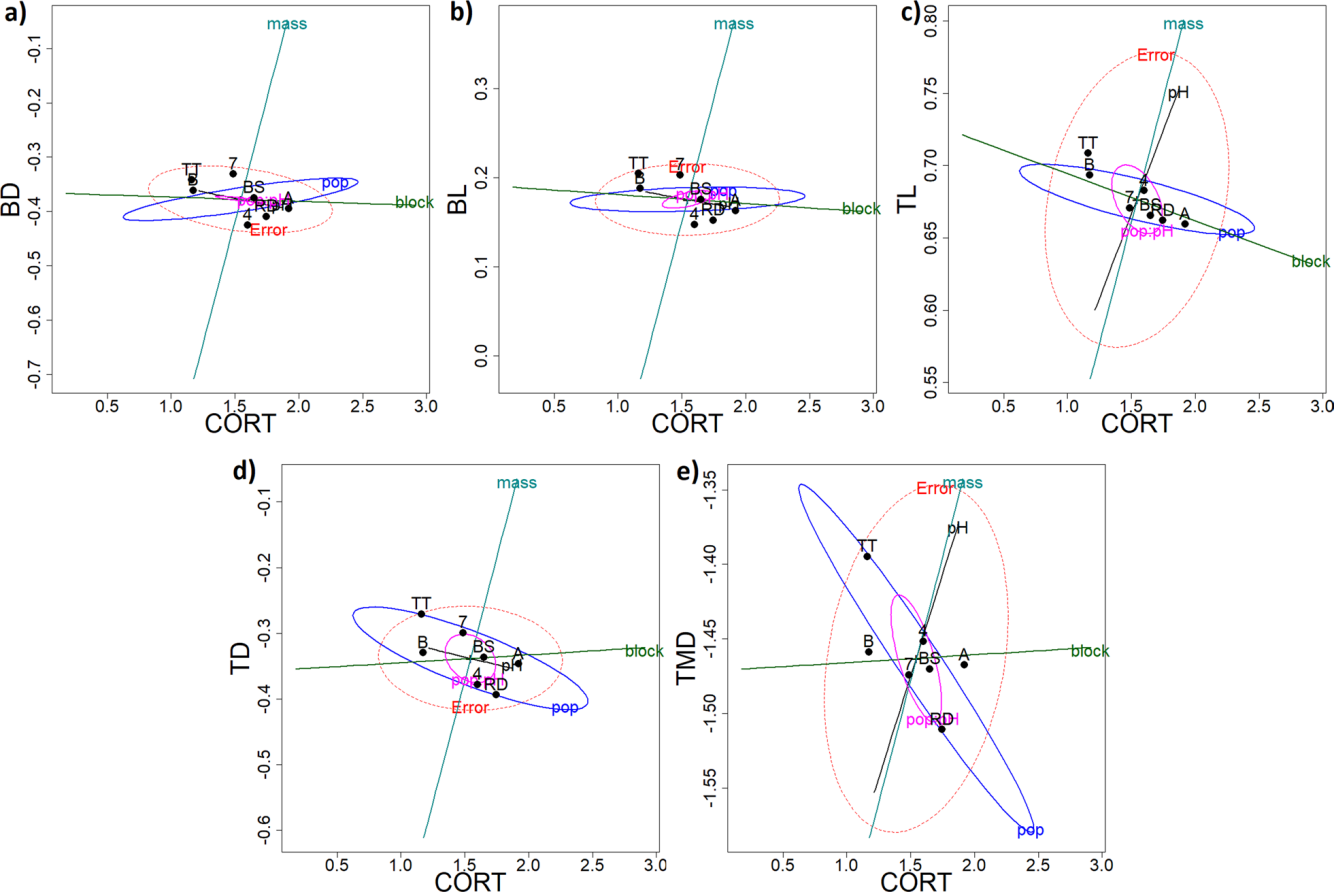
HE plots for CORT–trait associations from MANOVAs including CORT and morphology of *Rana arvalis* tadpoles at stage G32. Hypothesis ellipsoids that are outside of the Error ellipse (in red) reflect significant effects. The ellipses depicted are pop = population, ph = pH treatment, pop:ph = Population-pH treatment interaction, mass = tadpole mass, block = rearing Block. Solid symbols indicate fixed effect means for population (TT: Acid origin, BS: Intermediate origin, RD: Neutral origin), pH treatment (7: Neutral treatment, 4: Acid treatment) and Block (**A** morning sampling/warmer, **B** afternoon sampling/colder)

Based on the HE plots, there was a subtle negative relationship between CORT and tail length across blocks: tadpoles with higher CORT (A block) had shorter tails than tadpoles with lower CORT (B block) (Fig. 5 and Additional file 2: Fig. 2.3). There was a subtle negative relationship between CORT and both tail length and tail depth: tadpoles with higher CORT (RD) had shorter and shallower tails. The relationship between CORT and tail muscle depth was strongly associated to population: tadpoles with higher CORT levels (RD) had shallower tail muscles compared to those with lower CORT levels (TT). No other morphological traits were associated with variation in CORT.

**Fig. 6:**
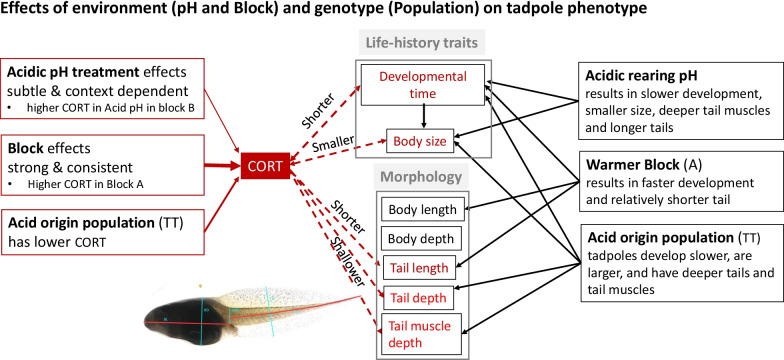
A schematic presentation of key findings on G32 tadpoles of *R. arvalis*. On the left, effects of fixed factors (pH treatment, rearing/sampling Block and Population) on CORT, as well as associations that were statistically significant between tadpole CORT levels and life-history and morphology traits. On the right, overview of effects of pH treatment, Block and Population on tadpole life-history and morphology traits. As life-history CORT—trait associations can be bi-directional (CORT affects trait, trait variation determines CORT), they are indicated by double headed arrows. Only effects that were statistically tested and significant are indicated. CORT—trait associations are highlighted in red

In summary, we found among population divergence in multivariate space for CORT, life-history traits and morphology of *R. arvalis* tadpoles (see Fig. 6). Specifically, tadpoles from an acid origin population (TT) had lower CORT levels, developed slower and were larger, and had relatively deeper tails and tail muscles during the mid-larval stage G32 relative to the neutral and intermediate origin population (RD and BS). Many effects were strongly affected by block effects, indicating strong phenotypic plasticity. Most intriguingly, we found negative associations between CORT levels and developmental time (at G32, G38 and G42), as well as CORT and tadpole tail length (at G32), across the rearing blocks: tadpoles from the A block had higher CORT levels and developed faster across all three developmental stages, they were also larger but had a shorter tail than those in the B block.

## Discussion

We found clear multivariate phenotypic divergence among tadpoles of three *R. arvalis* populations that were reared in acid *versus* neutral pH in the lab. In accordance with our previous studies [42], tadpoles from the acidic (TT) and the neutral pH origin population (RD) (two ends of an acidification gradient [42],) were more divergent, and tadpoles from the intermediate pH origin (BS) population were more variable or intermediate in their phenotype. Most intriguingly, we found among population divergence in CORT levels: TT tadpoles had, on average, lower CORT levels (especially at mid-larval stage G32) than RD tadpoles, with BS tadpoles being intermediate. Variation in CORT was, however, highly context dependent. As expected, CORT levels at metamorphosis (G42) were much higher than during mid-larval stages (G32 and G38). However, the effects of pH treatment on CORT were weak: only G32 tadpoles in one of the two rearing Blocks (B block) showed higher CORT levels in the Acid than the Neutral treatment. In contrast, block effects were generally strong both on CORT and other tadpole traits, and can reflect variation due to circadian rhythm and/or temperature (discussed below). Finally, our multivariate analyses of CORT—trait associations showed that higher CORT levels were related to faster development (at all stages) and to relatively shorter and shallower tails and shallower tail muscles (at G32).

### Corticosterone

The most striking and novel finding in our study is divergence in CORT profiles between the three *R. arvalis* populations. Given common garden rearing (and individuals originating from multiple families within each population), these results suggest genetic divergence in CORT levels, although a contribution of maternal effects is also possible [e.g. 44, 46, see below]. The lower CORT levels of TT tadpoles indicate that acidity mediated selection may have favoured the general downregulation of CORT to reduce CORT induced costs under chronic stress [19, 21–25], as a so called “protective” mechanism [14]. Alternatively, it is possible that selection has favoured lower baseline CORT levels through selection on other traits, such as metabolic rate [47] rather than stress responses per se. For example, selection may have favoured lower metabolic rates (and therefore lower CORT) due to differential energetic demands in the TT population.

While phenotypic divergence in baseline and stress induced CORT levels is known between species [e.g. 14, 47, 48, 50–52], evidence for among population divergence within species is rather sparse (but see studies on stress selected lines in rainbow trout [53, 54], and birds (e.g. dark-eyed junco, [55]) and wild barn owls [56]). Our results highlight the need to study intraspecific genetic divergence in CORT levels. Importantly, as CORT levels are often used as “stress indicators” for wild populations [57–59], our results strongly suggest that accounting for potential genetically based population differences in CORT is needed to draw meaningful conclusions—and to aid conservation strategies—based on CORT assays in the wild.

Although our previous studies along this study gradient have shown repeatedly pH related phenotypic divergence in *R. arvalis* tadpoles in life-history and morphology traits (see references above), it is crucial to keep in mind that population divergence could arise via genetic drift and historical contingency rather than divergent natural selection [60–62]. However, Q_st_–F_st_ comparisons, jointly with quantitative genetic line crosses [43, 45], do show that previously observed larval trait divergence across this acidification gradient is likely due to natural selection rather than neutral processes. Our previous studies further show that this divergence between acid and neutral pH origin populations is a result of direct genetic and adaptive maternal effects [44]. However, the relative contribution of these different sources of variation on CORT is yet to be tested.

### Context dependency in CORT variation

In line with previous studies [e.g. 33, 50], CORT levels differed across the developmental stages with generally higher levels at the metamorphic climax (G42) compared to pre- (G32) and pro- (G38) metamorphosis. This is in accordance with CORT, in interaction with thyroid hormones, playing an essential role in amphibian metamorphosis [reviewed in 26, 33, 63]. We also found a rearing block effect on CORT at G32 and G38, confirming the contextual effect of sampling time and environmental influence [64]: baseline CORT levels in vertebrates peak typically briefly before the diel activity period of a species [65–68] due to metabolic demands of behaviours, such as foraging [69]. However, in our study, the likely circadian effect due to sampling time (i.e. morning A and afternoon B sampling blocks) was confounded with temperature differences between the rearing Blocks (relatively warmer A and colder B block). These temperature differences were subtle, but in ectotherms even subtle thermal variation over extended periods of time (here over several weeks) can affect metabolic activity and, subsequently, CORT levels and growth rates [70–72].

In contrast to our predictions, the Acid pH treatment did not have a consistent effect on CORT levels and neither was there evidence for Genotype-Environment interactions between populations (i.e. no acid pH treatment induced CORT variation between TT, BS and RD tadpoles). Acidic pH treatment led to higher CORT levels only at G32 and only in the B block. This is in contrast to studies on adults of the salamander *Ambystoma jeffersonianum* in the wild [30] and tadpoles of the Iberian Spadefoot toad *Pelobates cultripes* in the lab [73], which found increased CORT levels in acidic conditions. These inconsistencies across studies likely reflect the high context dependency of CORT responses. In our study, the increase in response to the Acid treatment in the B block could be due to the colder temperature in this block interacting via metabolic rates with pH (see also above). However, it is also worth noting that *R. arvalis* is a relatively acid tolerant species [reviewed in 40], and acidic pH may therefore be stressful only in certain contexts (e.g. in interaction with suboptimal temperatures or predators), and it is possible that some effects on CORT seen in laboratory conditions are relatively weak because other stressors, such as predators, toxic metals, or resource limitation are not present. Finally, the chronic exposure of tadpoles to acidity (here several weeks) could have led to a general down regulation of CORT (and return to baseline levels) to avoid detrimental effects of chronically elevated CORT [74, 75]. Hence, studies on CORT responses under stressor interactions, and short-term responses to acidic pH, would be informative.

### CORT and the multivariate phenotype

*Life history traits*—CORT has many metabolic functions, including lipid metabolism, growth, tissue repair, reproduction, and immune defense [19, 20]. To understand how environmental stress in general, and stress hormones in particular, could mediate organismal evolution [e.g. 3, 76], it is important to link variation in CORT to variation in fitness [8, 11, 77]. In this context, it is particularly essential to understand how CORT relates to life history traits [78]. We found that in *R. arvalis* tadpoles higher CORT levels were associated to faster development within all of the three developmental stages, and these effects were primarily driven by block and (for G42) population effects (i.e. higher CORT and faster development in the warmer A block and RD population). This is in contrast to previous studies for pre-metamorphic tadpoles (here: G32), where *slower* development at naturally and experimentally increased CORT levels has been found in *R. pipiens* [35, 36] and *R. temporaria* [34]. For pro-metamorphic stages (here: G38), however, *faster* development at increased CORT levels is generally found [reviewed in 26, 37]. It is possible that our observation of higher CORT levels being associated with faster development across the Blocks is a by-product of warmer conditions in the A block (as discussed above) [e.g. 79]. Higher temperature in block A could have led to the observed correlation by elevating metabolic activity, and thereby developmental rate, without a causal link from CORT to developmental time. However, as we also saw this relationship across populations (TT tadpoles had lower CORT and developed slower), it is possible that there is a genetic link between CORT and developmental rate.

The associations between CORT and other measured traits may also depend on whether CORT levels investigated reflect natural variation or are manipulated [80]. Glennemeier and Denver [36] exposed *R. pipiens* tadpoles to experimentally elevated CORT levels, but within a natural range, and found slower growth and development and increased tail muscle depth, indicating a causal role for CORT for these traits. Elevated CORT levels could increase individual metabolism, thereby releasing energy to developmental processes and faster development [80], though possibly at the cost of reduced size. Extending this thought, mildly stressed tadpoles may benefit from elevated CORT levels by being able to metamorphose earlier and thereby escaping stressful conditions (such as drying ponds or predation; [7, 81, 82]). This hypothesis is also supported by findings on *P. cultripes* tadpoles and other species, which have higher CORT levels and speed up development when exposed to low water levels [7, 50, 81, 82]. Jointly these studies suggest that the relationship between CORT and developmental time is species and context specific.

Overall, patterns in life-history trait divergence in our study are comparable with previous studies on this system: acidic environmental conditions increase developmental time and reduce size of tadpoles and metamorphs, but when reared in common garden in the lab individuals from Acid origin populations (of which the population TT is the most extreme along our study gradient) develop slower but are larger than individuals from Neutral origin populations [42, 43]. In an earlier study, the negative effect of rearing acidity on developmental time to metamorphosis was stronger in Neutral origin populations [42]—suggesting that the differential effects of acidity on size at metamorphosis (a key fitness trait in amphibians, [83]) are mediated via developmental rates. Jointly, our results indicate divergence between *R. arvalis* populations in pH related effects on developmental time—possibly mediated by variation in CORT.

*Morphology*—Several studies suggest that CORT influences morphology, such as body length or tail depth, in tadpoles [e.g. 30, 31, 36]. In line with our previous study [41], we found morphological divergence between populations in multivariate space, with TT tadpoles having relatively deeper tails and tail muscles, especially in the acidic pH treatment. Deeper tails and tail muscles are key anti-predator traits in tadpoles and tail depth correlated with reduced predation risk in Acid origin tadpoles in our system [41]. However, here we found that higher CORT levels were associated with relatively shorter and shallower tails (Block and Population effect) [31] and shallower tail muscles (Population effect). Hence, these results indicate that CORT has the potential to influence some aspects of tadpole morphology, but experimental manipulations are clearly needed to infer causality between CORT, trait variation and fitness [84].

## Conclusion

We found strong context dependency of CORT levels and CORT-trait associations across three divergent *R. arvalis* populations. Our study suggests that CORT may especially affect tadpole developmental time and tail morphology (relative tail length, tail depth and tail muscle depth). In contrast to our predictions, however, we found no consistent evidence for elevated CORT under chronic acid stress. Instead, our study indicates divergent selection on baseline or chronic stress induced CORT levels along the acidification gradient. In addition to indicating the potential for natural selection to operate on physiological processes, this finding is crucial from an applied perspective: variation in baseline and/or chronic stress induced CORT levels among genotypes needs to be considered when using CORT levels as stress indicators in the wild [57–59].

This study is a first step towards shedding light on the potential role of CORT as a mediator of multi-trait divergence along environmental stress gradients, and manipulative studies are clearly needed to test the causal link between CORT, multivariate phenotype and fitness. Importantly, as natural populations typically face several natural and anthropogenic stressors in the wild, studies exposing tadpoles to multiple stressors in the laboratory or in mesocosms—jointly with experimental CORT manipulations, would allow investigations of potential fitness trade-offs and mediators of adaptation.

## Methods

### Study system

*Rana arvalis* occurs in northern, central and eastern Europe and western Siberia [85]. It breeds in ponds and small lakes at a broad range of pHs (pH 4 to 8) [85]. Reproduction takes place in spring soon after ice melt and the females lay a single clutch of 500–1500 eggs/breeding season [85, 86]. Depending on environmental conditions, development from fertilization takes approximately 10–12 days to hatching and 2–3 months to metamorphosis [43, 85].

### Study design

#### Experimental design

We studied three populations of *R. arvalis*: Tottatjärn (TT)—an acid pH origin population (breeding pond pH 4.0), Bergsjörn (BS)—an intermediate pH origin population (breeding pond pH 6.1), and Rud (RD)—a neutral pH origin population (breeding pond pH 7.0). These belong to well-studied populations along a pH gradient in South West Sweden [42]. We collected freshly fertilized eggs from the wild and reared tadpoles in the lab up to metamorphosis. Egg collection permits were obtained from the County board of Västra Götaland (permit number 522-6251-2017). All tadpole experiments and rearing from stage G25 on (start of exogenous feeding) require ethical permits. These permits were obtained from the ethical committee for animal experiments in Uppsala County (“Uppsala djurförsöksetiska nämnd”, permit number 5.8.18-01518/2017).

In each population, we collected ca. 50 freshly laid eggs (max. 2-cell stage, within ca. 30 min of egg laying) from each of eight clutches (= full-sib families) on the 3rd (TT), 5th (RD and BS) and 8th (RD and BS) of April 2017. The eggs were immediately placed in family specific groups to reconstituted soft water (RSW, see below), maintained cool (+ 4–7 °C, WAECO freezer and cooler) and transported to the laboratory facilities in Uppsala (Uppsala University), Sweden. Prior to the experiment, the embryos were reared in the lab in pH 7.5 RSW in groups of ca. 25 embryos in 0.9L PP plastic vials. Water for embryos was changed every few days to assure good water quality.

Experimental tadpoles were reared singly from G25 to G32, G38 or G42 [45]. From stage G25 on, the experimental design consisted of 3 Populations (TT, BS, RD) × 2 pH treatments (Acid and Neutral) × 3 developmental stages (G32, G38 and G42) × 8 families × 2 Blocks (A, B) (total N = 288). This means that each population—pH treatment combination, per Block and developmental stage, was represented by eight replicates (1 individual/family). Sample sizes were decided based on previous experiments using the same populations [42], whilst making the experiment logistically feasible (i.e. how many individuals could be sampled per time window per day). This is a factorial experiment, where hypothesis testing is based on comparisons of different population—pH treatment combinations  (i.e. comparisons are based on Neutral (benign) versus Acid (stressful) treatment and ‘handling’ controls are not relevant).

The two experimental Blocks (A and B) were based on a known temperature gradient in the room, with one replicate of each population-pH treatment combination in each block. These two Blocks were for logistic reasons sampled at different time points in a day, thereby bracketing biologically relevant context dependent variation in CORT and trait expression. The A block (mean temperature 15.9 ± 0.6 °C; range: 14.6–17.8 °C) was sampled in the morning, the B block (mean temperature 15.7 ± 0.6 °C; range: 13.7–17.1 °C) in the afternoon (see below).

#### Rearing conditions

The experiment was conducted in a walk-in climate room at 17 °C and 17L:7D light cycle. Once larvae reached G25 (exogenous feeding starts, [45]), they were randomly assigned to their respective pH treatment and placed individually to experimental containers in a randomly assigned location (location within each block was based on Excel randomization function) (see below). Tadpoles were reared individually in the Acid (target pH 4.3—with mean pH 4.8) or Neutral (target pH 7.5—mean pH 7.3) pH treatment from G25 to mid-larval stages G32, G38 or start of metamorphosis (G42, emergence of at least one front leg) following standard procedures [43], with some modifications (see details below). The tadpoles were reared in 0.9 L PP plastic vials, with 0.7 l treatment water, and equipped with a folded piece of non-transparent PP to provide shelter. Individual ID number, Population, pH treatment and Block was indicated on each sampling container to allow quick processing and reduce risk of error during maintenance and sampling.

We used reconstituted soft water (RSW; 48 mg/l NaHCO_3_, 30 mg/l CaSO_4_ × 2H_2_O, 61.4 mg/l MgSO_4_ × 7H_2_O, 2 mg/l KCl diluted in deionized water, [87]) throughout the experiment. Water was prepared in 204 l Nalgene tanks. The pH in the Acid treatment was adjusted by adding 1 M H2SO4. Both Acid and Neutral water was treated with peat pellets (Zoobest Gartenteich Torfpellets, ZB-01270; Acid treatment: 165 g/204 l, Neutral treatment:16.5 g/204 l) in a fine mesh bag to stabilize pH (Acid treatment), to account for peat presence (Neutral treatment), and to reflect natural occurrence of humic compounds in surface waters. The water stocks were always aerated and prepared a minimum of two days prior of usage to ensure dissolving of salts and stable pH. During the experiment, pH (Orion™ 3-Star pH Portable Meter & Orion™ ROSS Ultra™ Refillable pH/ATC Triode™ Combination Electrodes, Thermo Scientific™) and temperature (digital thermometer, Testo 108, EN 13,485, ± 0.5 °C) was measured in a subset of experimental vials before each water change. The pH mean ± SE in the Acid treatment was 4.82 ± 0.04 (A block) and 4.77 ± 0.04 (B block) and in the Neutral treatment 7.31 ± 0.01 (A block) and 7.29 ± 0.02 (B block). The pH was somewhat higher than target pH in the Acid treatment due to the addition of food. For water change, each individual tadpole was briefly placed in a wet, handheld dip net, water in the rearing container replaced and the tadpole gently returned to its respective container and provided with fresh food.

From G25 to G32, the tadpoles were fed ad libitum a mixture of finely ground organic parboiled spinach (Coop, Sweden) and organic spirulina powder (RenéeVoltaire, Sweden) (200 g spinach and 8.08 g spirulina and 10 ml of RSW). From G32 onwards, 0.5 g freeze dried *Tubifex* worms (Tubi Cubes, Tropical) were added to the mixture. This was done to account for differential nutritional demands of the developing tadpoles (i.e. optimal total protein content of ca. 35% protein at later larval stages; [88, 89]). Each tadpole received approx. 0.05 ml of the mixture at each water change during early developmental stages, and the amount was gradually increased to 0.3 ml as tadpoles grew. Water change and feeding took place every 2–3 days. Tadpoles were initially screened for developmental stages every four days and when approaching the desired sampling stage (G32, G38 or G42), daily. Each individual tadpole’s health and well-being was checked during each water change. If needed, animals were humanely sacrificed (N = 6 for this experiment) based on the following humane endpoints: animal was not eating, development was stunted or individuals showed signs of discomfort or neurological abnormalities (erratic movement, swimming in circles). As this occurred early in the experiment, individuals that had to be removed were replaced by “extra individuals” (N = 6) reared under the same conditions.

#### Response variables and sampling procedures

We measured whole-body CORT content, developmental time (days from G25 until G32, G38 or G42, respectively), tadpole mass (g) and morphology (body length, body depth, tail length, maximum tail depth, tail muscle depth, See Fig. 1). Mortality was assessed at each water change, but was low (3.13%, N = 9) and was not statistically analysed. Hence, a total of N = 282 individuals were included in data analyses. Since CORT profiles and tadpole morphology change over the course of development [26], samples were taken at early-mid larval stage G32 (pro-metamorphosis), late-mid larval stage G38 (pre-metamorphosis), and at metamorphosis G42 [45]. To determine developmental stage, individuals were visually inspected one day prior to sampling under a binocular microscope (Leica MZ6) for mid-larval stages, and with bare eye for onset of metamorphosis [45]. All sampling procedures were conducted in the walk-in rearing lab, but in an area physically separated from the rearing shelving. For logistic reasons, an individual that had reached a given sampling stage (G32, G38 or G42) was sampled 21–26 h after reaching a target stage. Likewise, tadpoles in block A were sampled in a haphazard order in the morning, whereas tadpoles in block B were sampled in a haphazard order in the afternoon. Although this temporally structured sampling of the two Blocks confounds potential effects of temperature and circadian rhythm [68] (see discussion), this approach was taken to maximize physiological variation and to standardize variation across population and treatments, whilst making the experiment logistically feasible.

Because whole body CORT cannot be sampled without sacrificing the tadpoles, tadpoles at a given stage were deeply narcotized in 2 g/l MS222 (Ethyl-3-aminobenzoate-methanesulfonate, Sigma Aldrich, E10521) dissolved in buffered RSW until they did not respond to external stimuli, a commonly used method for narcotizing amphibians [90, 91]. Although MS222 may affect CORT levels (e.g. [92–94]) this should not affect our inferences as all animals were handled the same way. Each tadpole was subsequently sacrificed via snap freezing at -80 °C in conjunction to CORT sampling (see below). Animals were then gently dry blotted on tissue paper and weighed for total wet mass with a digital balance (VWR, SE 203-LR) to nearest of 0.001 mg. For measurements of morphology (Fig. 1), a digital image (Digital camera: OLYMPUS, CAMEDIA C-5060 Wide Zoom, 5.1 Megapixels;lense: hama UV390, 4X OLYMPUS WIDE ZOOM LENS ED) was taken on each individual by placing the tadpole on its side on a Petri dish, equipped with millimeter paper as a scale. A small part of the tip of the tail was cut and placed in 95% EtOH to allow later genetic analyses, and the deeply anaesthetized tadpole was snap frozen in a sterile 3.5 ml PP vial (60.549.001, Sarstedt) marked with ID number and population treatment combination, in liquid nitrogen for CORT analyses. CORT samples were stored at − 80 °C until extraction.

#### Tadpole morphology

Tadpole morphology (Fig. 1) was measured from the digital images using ImageJ (imagej.net, year 2017) following previously described procedures [41]. Each picture included a number and letter code, the meaning of which was not explained in detail to the person (N. Tardent and N. Weissert) that was measuring the pictures. The code was used to allow for double control of individual ID and treatment combination. Morphology was measured at G32 and G38 as body length, body depth, tail length, maximum tail depth and tail muscle depth. As tadpole morphology at G32 (mid-larval stage) has been shown to be most divergent in previous studies, is closely related to tadpole fitness [41], and showed most variation in multivariate space (Additional file 3) we concentrated on G32 for analyses of morphology.

#### Hormonal analyses

Organic phase extraction with Ethyl acetate was conducted and standard Enzyme Immuno Assays (EIA, Arbor assays) hormonal assessments (adapted from [95]) were carried out with a plate reader (Molecular devices, SpectraMax 190). The hormonal assay methods were first validated by determining stress metabolites using CORT manipulation pilot studies, tissue comparisons and by comparing EIA results with mass spectrometry results (Additional file 4). These data confirmed that corticosterone (CORT) is the main biologically relevant glucocorticoid in *R. arvalis* tadpoles, and that whole body samples are the most robust and logistical feasible tissue to sample and reflect and integrative measure of individual hormonal levels over time.

CORT extraction was conducted at Uppsala University, Sweden. For extraction, samples were defrosted in blocks of developmental sampling stages and homogenized in haphazard order using an Ultraturrax (TP18/10) for 30 s. The tissue ruptor was cleaned with 99% EtOH and ddH20 between samples. 0.80–0.85 g of each of the homogenized sample was pipetted (using filter tips) into a sterile 2 ml PP screw tube (Sarstedt, 72.693.005) and 1500 µl of Ethyl acetate (99.8%, Sigma Aldrich, 270989) was added. Samples were mixed using a VWR Vortex for 30 s and subsequently transferred to 4 °C and shaken on a plate shaker for 30 min (IKA MSR 3 digital). The samples were centrifuged at 5000 Rpm (VWR, Micro Star 17) for 15 min. The resulting supernatant (approx. 1450 µl) was pipetted (using filter tips) into safe lock tubes (2 ml, Eppendorf, PP) and stored at − 20 °C. For extraction, samples were thereafter evaporated in a SpeedVac at 45 °C (SpeedVac plus, SC110A attached to Savant, Gel Pump GP110). Upon extraction, all samples were filled up with a stream of N2 to prevent oxidation, sealed with Parafilm and transported dry at room temperature to the Swiss Federal Institute of Aquatic Science and Technology (EAWAG) in Duebendorf, Switzerland. The samples were then reconstituted in 115 µl assay buffer (Arbor Assays Detect X Corticosterone Enzyme Immunoassay Kit, K014-H1/H5) and 5 µl 99% EtOH, vortexed and stored at − 20 °C for later EIA analyses.

The EIA was conducted following the Arbor Assays Detect X Corticosterone Enzyme Immunoassay Kit (K014-H1/H5) instructions. Standard curve was adapted due to the relatively low CORT concentration of some samples by using a concentration range from 5000 to 39 pg/ml. Samples were chosen haphazardly within each developmental stage and run in duplicates as there was not enough extracted sample material for more technical replicates (i.e. insufficient concentrations for further testing and diluting). To minimize pipetting errors and plate contamination, we followed standard endocrinological methods of pipetting duplicates next to each other (e.g. position C5 and D5) (personal communication, W. Goymann) which, however, comes at a cost of possible well-to-well contamination and statistical non-independence. The washing step in the protocol was performed using a plate washer (BioTek, ELx50). Optical density (OD) of each sample was measured at 450 nm with a plate reader (Molecular devices, SpectraMax 190). The OD was transformed to CORT concentrations (pg/ml) using the provided Arbor Assay software (https://www.myassays.com/). The software estimates the sample concentration by interpolation to a standard curve (four parameters). The manufacturer gives a sensitivity for the used assay of 18.6 pg/ml and a detection limit of 16.9 pg/ml. Cross reactivities were tested by the manufacturer for several substances and are listed in the kit manual (Arbor Assays Detect X Corticosterone Enzyme Immunoassay Kit, K014-H1/H5). Except for Desoxycorticosterone (12.3%) cross reactivities were all below 0.8%. Plate intra- and inter-assay coefficients of variation were calculated using standards (low- and high-level group) run on each plate. Intra-assay coefficient of variation on average was 10.56% (low: 15.58%, high: 5.54%). Inter-assay coefficient of variation on average was 7.81% (low: 12.08%, high: 3.54%). The average coefficient of variation of duplicates run over all plates was 9.60%. CORT concentrations were corrected for mg of extracted tissue and µl (50 µl) of the sample used for each well resulting in CORT concentrations of pg/mg tadpole tissue.

#### Statistical analyses

All statistical analyses were conducted in Rstudio (Version 1.1.383 R 3.6.0 (2019-04-26) & Version 1.2.5033 R3.6.2). Statistical analyses were conducted using a series of univariate and multivariate models. Analyses were done on log-transformed response variables in order to reach normality (where relevant) and to assure all traits were on similar scales in multivariate analyses. Data was checked for normality before and after log transformation. In all linear models, normality was visually assessed using QQ plots and by checking the distribution of residuals. All data sets were analysed by J. Mausbach and had information on population treatment combination. In a few cases, individuals appeared as outliers in some traits (one extreme value in CORT and TD). These individuals were retained in the analyses because they did not influence statistical significance and were more likely to represent biologically extreme values than measurement error or other unwanted variation.

### Univariate linear models

Data was analysed using linear models (ANOVA Type III in nlme package using options(contrast = c("contr.sum","contr.poly")) and drop1(model,. ~ .,test = "F") [96] or the “car” package [97]). The full models included fixed factors of Population (3 levels), pH treatment (2 levels), Developmental stage (3 levels), Block (2 levels) and relevant two- to four-way interactions. We always started with a full model containing all fixed effect interactions. To then reduce model complexity, we sequentially removed non-significant interactions (*P* ≥ 0.05, starting with four-way interactions) using backwards selection of the linear models, with always maintaining all main effects and experimentally meaningful interactions in the model. For example, the Population x pH treatment interaction was always retained as this tested a key hypothesis and was an essential part of the fully factorial study design. Next, CORT was analysed within each of the developmental stages (G32, G38 and G42) separately. In these models, main effects of Population, pH, Block and their interactions were included. Where relevant and not statistically confounded, tadpole mass was included as a covariate. Non-significant covariate interactions and covariate main effects were sequentially removed and only final models are reported here. As the three study populations differ substantially in larval mass (Table 1 and Additional file 1: Table 1.3), and population and mass would be statistically confounded, mass was not added as a covariate in final univariate statistical models on CORT. Instead, to test whether tadpole size affected CORT levels models with log(mass) as covariate were run within each study population (Additional file 1: Table 1.2). This ANCOVA was done only within the G32 stage. We report Means ± SE of the data and tests for relevant pairwise differences in LSmeans using post hoc Tukey tests (R package: lsmean, FSA, ggplot2).

### Multivariate phenotype

#### CORT and Life history

In order to assess covariance between CORT, developmental time (days from G25 until G32, G38 or G42) and tadpole mass, MANOVAs were run within G32, G38 and G42 stages. These analyses included log(CORT), log(developmental time) and log(mass) as response variables. Fixed factors of Population (3 levels), pH treatment (2 levels) and Block (2 levels), and their two- to three-way interactions, were included as predictors. We always started with a full model containing all fixed effect interactions and reduced model complexity by sequentially removing non-significant interactions (*P* ≥ 0.05, starting with four-way interactions) using backwards selection. Only final models are reported here.

For final models, we calculated the partial variance eta^2^ [98], using the heplot package in R [99, 100]. We report Wilk’s, Pillai’s and Hotteling Lawley’s eta^2^ with ranking of all partial variances. However, we only present test statistics for Wilk’s tests (MANOVA type III, contrasts = list(topic = contr.sum, sys = contr.sum)). For visual presentation of MANOVAs we used the package heplot (MANOVA type III) in R [99, 100] that plots ellipsoids of hypotheses (H) and the error (E) of a given model. Significance is indicated by H ellipsoids reaching out of the E ellipsoid [99, 100]. The HE plots were run both on canonical models (candisc package [101]), to visualize the overall MANOVA results in one plot, and as simple HE plots derived from the MANOVA. As we were specifically interested in the mid-larval stage G32, the canonical analyses were conducted only for G32 tadpoles. All MANOVAs were followed by univariate linear models (ANOVA type III, using options(contrast = c("contr.sum","contr.poly")) and drop1(model,. ~ .,test = "F")). We report means ± SE of the univariate data and test for relevant pairwise differences of LSmeans using post hoc Tukey tests (R package: lsmean, FSA, ggplot2 [102–104]).

#### Morphology and CORT relationship

*Visual representation morphology and CORT—*To assess covariance visually across the full phenotype at mid-larval stages G32 and G38, a Discriminant Analysis of Principle Components (DAPC) was conducted (R adegenet package 2.0, [105, 106]). In this multivariate statistical approach, the variance of the data is partitioned into a between-group and within-group component, in order to maximize the discrimination between the groups [105]. The data was first analysed using a principal component analysis (PCA) and afterwards clusters identified using discriminant analysis (DA) [105].

In our data set, combinations of developmental stage (G32 and G38), Population (TT, BS, RD), pH treatment (Acid and Neutral) and Block (A and B) were used to assign individuals into groups. These analyses were conducted sequentially for two DAPCs:

I. The 1st DAPC included log CORT, tail length, tail depth, tail muscle depth, body depth and body length at G32 and G38.
II. The 2nd DAPC included log CORT, tail length, tail depth, tail muscle depth, body depth and body length at G32.

For all DAPCs, contributions of the Loadings (e.g. LD1, LD2) were calculated by dividing the respective LD through the sum of “eigenvalues”. Only LD1 and LD2 were considered further as the data were sufficiently described by these two (> 80%) and all variable contributions that were above 10% for those LDs are reported. An ordination and loading plot was used to illustrate the grouping and most relevant contribution of variables.

*Multivariate tadpole morphology and CORT at G32*—In order to assess the covariation between CORT and multivariate morphology (body depth, body length, tail length, tail depth, tail muscle depth), a MANCOVA was run at G32. In these models all traits were log transformed. Fixed effects of Population (3 levels), pH treatment (2 levels) and Block (2 levels) as main effects, log(mass) as a covariate and relevant two to four-way interactions were included as explanatory variables. Model selection, partitioning of variance, and univariate model testing and visualization, was conducted as for the CORT—life history MANOVA (see above). Visual representation of the MANCOVA conducted with HE plots using the heplot package and manual [99, 107].

## Supplementary Information

**Additional file 1:** Additional Results for univariate and multivariate AN(C)OVAs

**Additional file 2:** Canonical models and additional HE plots of MAN(C)OVAs

**Additional file 3:** Additional DAPC results

**Additional file 4:** Validating the hormonal sampling and assay methods

## Data Availability

The datasets generated and/or analysed during the current study are available upon publication at Dryad. https://doi.org/10.5061/dryad.tx95x69zp.

## Abbreviations

CORT: Corticosterone
EIA: Enzyme Immuno Assay
TT: Tottatjärn (acid origin population, breeding pond pH 4.0)
BS: Bergsjön (intermediate origin population, breeding pond pH 6.1)
RD: Rud (neutral origin population, breeding pond pH 7.0)
4 Acid: pH treatment (target pH 4.3)
7 Neutral: pH treatment (target pH 7.5)
BL: Body length
BD: Body depth
TL: Tail length
TD: Maximum tail depth
TMD: Tail muscle depth
RSW: Reconstituted soft water
temp.: Temperature
dev. Time: Developmental time (days from G25)
treatm.: Treatment
