# Correction to: Context dependent variation in corticosterone and phenotypic divergence of *Rana arvalis* populations along an acidification gradient

**DOI:** 10.1186/s12862-022-01982-2

**Published:** 2022-03-07

**Authors:** Jelena Mausbach, Anssi Laurila, Katja Räsänen

**Affiliations:** 1 Department of Aquatic Ecology, Eawag, Ueberlandstrasse 133, 8600 Duebendorf, Switzerland; 2 Institute of Integrative Biology, ETH Zurich, Universitätstrasse 16, 8092 Zurich, Switzerland; 3 Animal Ecology/Department of Ecology and Genetics, Evolutionary Biology Centre, Uppsala University, Norbyvägen 18D, 75236 Uppsala, Sweden; 4 Department of Biological and Environmental Science, University of Jyväskylä, Survontie 9C, 40014 Jyväskylä, Finland

## Correction to: BMC Ecology and Evolution (2022) 22:11 10.1186/s12862-022-01967-1

Following the publication of the original article [1], we were notified that the authors' first and last names have been swapped.

Originally published names: Mausbach Jelena, Laurila Anssi and Räsänen Katja.

Corrected names: Jelena Mausbach, Anssi Laurila and Katja Räsänen.

The original article has been corrected.
