# Supplementary material for: Context dependent variation in corticosterone and phenotypic divergence of Rana arvalis populations along an acidification gradient
Source: BMC Ecol Evol. 2022 Feb 5;22:11. doi: 10.1186/s12862-022-01967-1 (PMC8818180; doi:10.1186/s12862-022-01967-1)
Supplement: Supplementary file 1 — Additional file 1: Additional Results for univariate and multivariate AN(C)OVAs [file 12862_2022_1967_MOESM1_ESM.docx]

**Additional file 1: Additional Results for univariate and multivariate AN(C)OVAs**

**Additional Table 1.1.** Univariate linear models on CORT, developmental time and mass across G32, G38 and G42

**Additional Table 1.2.** Univariate linear models on CORT of G32 tadpoles within RD, BS and TT populations.

**Additional Table 1.3.** Multivariate AN(C)OVAs of CORT and life history traits and CORT and morphological traits at G32, G38 and G42, and CORT and morphology at G32

**Summary of results from univariate ANOVAs** (derived from the MANOVAs in Table 1.3) and **Additional Table 1.4** for CORT and life-history *A)* at G32, *B)* at G38 and *C)* G42 stages and *D)* CORT – morphology at G32.

**Additional Figure 1.1.** LS means ± SE of morphological traits corrected for tadpole mass for G32 tadpoles.

**Additional Table 1.1.** Univariate linear models on log(CORT), log(developmental time) (days) and log(mass) (g) across three developmental stages (G32, G38 and G42) of *Rana arvalis* tadpoles from three populations (RD: neutral pH origin, BS: intermediate pH origin and TT: acidic pH origin), reared in two pH treatments (Acid and Neutral) and two Blocks (A: morning/warmer, B: afternoon/colder). The data represent rearing from early larval stage (G25) to the respective developmental stage. These are final models following removal of non-significant three or four way interactions. Statistically significant effects (p < 0.05) are shown in bold.

| Factors | *Traits* | | | | | | | | |
| --- | --- | --- | --- | --- | --- | --- | --- | --- | --- |
|  | 1. *log(CORT)* | | | 1. *log(dev.time)* | | | 1. *log(mass)* | | |
|  | *df* | *F* | *P* | *df* | *F* | *P* | *df* | *F* | *p* |
| Population | 2 | 14.60 | **<0.001** | 2 | 5.58 | **0.004** | 2 | 16.73 | **<0.001** |
| pH treatment | 1 | 1.09 | 0.297 | 1 | 28.08 | **<0.001** | 1 | 28.88 | **<0.001** |
| Block | 1 | 125.77 | **<0.001** | 1 | 36.83 | **<0.001** | 1 | 2.93 | 0.088 |
| Stage | 2 | 660.97 | **<0.001** | 2 | 913.63 | **<0.001** | 2 | 404.49 | **<0.001** |
| Population x pH | 2 | 0.44 | 0.644 | 2 | 0.31 | 0.732 | 2 | 2.66 | 0.072 |
| pH x Block | 1 | 0.37 | 0.543 | - | - | - | - | - | - |
| Population x Stage | 4 | 3.45 | **0.009** | 4 | 2.81 | **0.026** | **-** | **-** | **-** |
| pH x Stage | 2 | 0.85 | 0.429 | 2 | 8.58 | **<0.001** | 2 | 9.83 | **0.001** |
| Block x Stage | 2 | 20.63 | **<0.001** | **-** | **3.29** | **0.039** | - | **-** | **-** |
| pH x Block x Stage | 2 | 4.89 | **0.008** | **-** | **-** | **-** | - | **-** | **-** |
| Residual SE and (*df*) | 0.35 (254) | | | 0.06 (270) | | | 0.16 (275) | | |

**Additional Table 1.2.** Results of univariate linear models on log(CORT) of G32 tadpoles within three *Rana arvalis* populations (RD: neutral pH origin, BS: intermediate pH origin and TT: acid pH origin), reared in two pH treatments (Acid ‘4’ and Neutral ‘7’) and two blocks (A: morning/warmer, B: afternoon/colder). In these models log(mass) was included as a covariate. Significant effects (p< 0.05) are shown in bold. Separate population-specific models at G32 showed that tadpole mass did not affect CORT.

|  | ***Population*** | | | | | | | | |
| --- | --- | --- | --- | --- | --- | --- | --- | --- | --- |
|  | *RD* | | | *BS* | | | *TT* | | |
| **Factors** | *df* | *F* | *P* | *df* | *F* | *P* | *df* | *F* | *p* |
| pH | 1 | 2.45 | 0.129 | 1 | 0.29 | 0.593 | 1 | 0.72 | 0.404 |
| block | 1 | 34.52 | **<0.001** | 1 | 10.90 | **0.003** | 1 | 16.98 | **<0.001** |
| log(mass) | 1 | 0.73 | 0.401 | 1 | 1.15 | 0.292 | 1 | 1.97 | 0.172 |
| Residual  SE (*df)* | 0.39 (28) | | | 0.47 (27) | | | 0.55 (25) | | |

**Additional Table 1.3.** Results of multivariate AN(C)OVAs (MANOVAs and MANCOVAs) on A-C) CORT and life-history at three developmental stages (G32, G38 and G42) and D) CORT and morphology at G32 in *Rana arvalis* tadpoles. Analyses present data from three (RD: neutral pH origin, BS: intermediate pH origin and TT: acid pH origin) populations, reared in two pH treatments (Acid ‘4’ and Neutral ‘7’) and two blocks (A: morning/warmer, B: afternoon/colder). Significant effects (p< 0.05) are shown in bold. All traits were log transformed for analyses. Partial variance (eta^2^) and ranking (rank 1 = strongest contribution) is shown for three different test statistics (Wilk’s lambda, Hotteling-Lawley’s and Pillai’s). Eta^2^ values show the relative contribution of the different predictors. See Figure 2 in main text for visualization and for N of CORT, mass and developmental time. N = 8 except for RD4B, TT4B, TT7B (N= 7), TT4A (N = 6) and TT7A (N = 5).

|  | *df* | | *Wilk’s test stat* | | *approx. F* | | *ndf, ddf* | | *P* | | *Eta^2^ & rank* | | | | |
| --- | --- | --- | --- | --- | --- | --- | --- | --- | --- | --- | --- | --- | --- | --- | --- |
|  |  |  |  |  |  |  |  |  |  |  | *Wilk’s lambda* | | *H.-La.* | | *Pillai* |
| 1. *G32 - CORT, developmental time, mass* | | | | | | | | | | | | | | | |
| *Population* | 2 | 0.67 | | 6.2 | | 6, 166 | | **<0.001** | | 0.18 3 | | 0.18 3 | | 0.18 3 | |
| *pH* | 1 | 0.79 | | 7.4 | | 3, 83 | | **<0.001** | | 0.21 2 | | 0.21 2 | | 0.21 2 | |
| *Block* | 1 | 0.46 | | 32.7 | | 3, 83 | | **<0.001** | | 0.54 1 | | 0.54 1 | | 0.54 1 | |
| *Pop x pH* | 2 | 0.97 | | 0.4 | | 6, 166 | | 0.875 | | 0.01 4 | | 0.01 4 | | 0.01 4 | |
| 1. *G38 - CORT, developmental time, mass* | | | | | | | | | | | | | | | |
| *Population* | 2 | 0.68 | | 6.0 | | 6, 162 | | **<0.001** | | 0.18 3 | | 0.17 3 | | 0.17 3 | |
| *pH* | 1 | 0.54 | | 23.0 | | 3, 81 | | **<0.001** | | 0.46 2 | | 0.46 2 | | 0.46 2 | |
| *Block* | 1 | 0.34 | | 52.0 | | 3, 81 | | **<0.001** | | 0.66 1 | | 0.67 1 | | 0.66 1 | |
| *Pop x pH* | 2 | 0.86 | | 2.0 | | 6, 162 | | **0.049** | | 0.07 4 | | 0.08 4 | | 0.07 4 | |
| 1. *G42 - CORT, developmental time, mass* | | | | | | | | | | | | | | | |
| *Population* | 2 | 0.66 | | 6.0 | | 6, 164 | | **<0.001** | | 0.19 1 | | 0.20 1 | | 0.18 1 | |
| *pH* | 1 | 0.95 | | 2.0 | | 3, 82 | | 0.205 | | 0.05 3 | | 0.05 3 | | 0.05 3 | |
| *Block* | 1 | 0.83 | | 6.0 | | 3, 82 | | **0.001** | | 0.17 2 | | 0.17 2 | | 0.17 2 | |
| *Pop x pH* | 2 | 0.95 | | 1.0 | | 6, 164 | | 0.667 | | 0.02 4 | | 0.02 4 | | 0.02 4 | |
| 1. *G32 – CORT, morphology (body depth, body length, tail length, tail depth, tail muscle depth)* | | | | | | | | | | | | | | | |
| *Pop* | 2 | 0.50 | | 5.47 | | 12,146 | | **<0.001** | | 0.30 3 | | 0.31 3 | | 0.28 3 | |
| *pH* | 1 | 0.79 | | 3.27 | | 6,74 | | **0.007** | | 0.21 4 | | 0.21 4 | | 0.21 4 | |
| *Block* | 1 | 0.55 | | 10.11 | | 6,74 | | **<0.001** | | 0.45 2 | | 0.45 2 | | 0.45 2 | |
| *log(mass)* | 1 | 0.06 | | 203.92 | | 6,74 | | **<0.001** | | 0.94 1 | | 0.94 1 | | 0.94 1 | |
| *pop x pH* | 2 | 0.88 | | 0.78 | | 12,146 | | 0.667 | | 0.06 5 | | 0.06 5 | | 0.06 5 | |

**Summary of results from univariate ANOVAs (Additional Table 1.4) derived from the MANOVAs (Additional Table 1.3)**

*Life history traits -* Univariate ANOVAs for G32 indicated strong and significant block effects on CORT and developmental time (days from G25), significant pH treatment effects on developmental time and mass, and significant population main effects on CORT, developmental time and mass (Add. Table 1.4). Univariate ANOVAs for G38 found strong and significant block effects on CORT and developmental time, as well as significant pH treatment and population effects on developmental time and mass (Add. Table 1.4). Univariate ANOVAs for G42 revealed that block primarily affected developmental time (earlier metamorphosis in A block) and population affected tadpole mass (TT, acidic pH origin) individuals were larger at metamorphosis than RD (neutral pH origin) and BS (intermediate pH origin) and developmental time (Add. Table 1.4).

*Morphological traits -* There was a significant block effect on CORT, but not on any of the size corrected morphological traits (Add. Table 1.4). The significant population main effect arose as TT (acidic pH origin) tadpoles, on average, had deeper tails and tail muscles (for a given size) than RD (neutral pH origin) tadpoles (see Add. Figure 1.1). The pH affect arose as tadpoles overall had longer tails but deeper tail muscles when reared in acidic conditions (see Add. Figure 1.1).

**Additional Table 1.4**. Results of univariate linear models (ANOVAs) derived from MANOVAs for CORT and life-history traits *A)* at G32, *B)* at G38 and *C)* G42 stages and *D)* CORT – morphology at G32 in *Rana arvalis* tadpoles from three *Rana arvalis* populations (RD: neutral pH origin, BS: intermediate pH origin and TT: acidic pH origin), reared in two pH treatments (Acid ‘4’ and Neutral ‘7’) and two blocks (A: morning/warmer, B: afternoon/colder). Significant effects (p< 0.05) are shown in **bold**.

| 1. *CORT - life history at G32* | | | | | | | | | |
| --- | --- | --- | --- | --- | --- | --- | --- | --- | --- |
|  | *log(CORT)* | | | *log(developmental time)* | | | *log(mass)* | | |
| ***Factors*** | *Df* | *F* | *p* | *Df* | *F* | *P* | *df* | *F* | *p* |
| *Population* | 2 | 10.11 | **<0.001** | 2 | 6.67 | **0.001** | 2 | 6.09 | **0.003** |
| *pH* | 1 | 0.79 | 0.376 | 1 | 33.40 | **<0.001** | 1 | 20.86 | **<0.001** |
| *Block* | 1 | 56.08 | **<0.001** | 1 | 27.32 | **<0.001** | 1 | 1.20 | 0.276 |
| *pop x pH* | 2 | 0.33 | 0.723 | 2 | 0.68 | 0.511 | 2 | 0.39 | 0.679 |
| *Residual SE (df)* | 0.47 (86) | | | 0.07 (89) | | | 0.20 (88) | | |
| 1. *CORT - life history at G38* | | | | | | | | | |
|  | *log(CORT)* | | | *log(developmental time)* | | | *log(mass)* | | |
| ***Factors*** | *Df* | *F* | *p* | *Df* | *F* | *P* | *df* | *F* | *p* |
| *Population* | 2 | 2.42 | 0.095 | 2 | 11.38 | **<0.001** | 2 | 4.51 | **0.014** |
| *pH* | 1 | 0.037 | 0.546 | 1 | 46.20 | **<0.001** | 1 | 37.35 | **<0.001** |
| *Block* | 1 | 152.51 | **<0.001** | 1 | 9.09 | **0.003** | 1 | 0.53 | 0.468 |
| *pop x pH* | 2 | 1.36 | 0.262 | 2 | 1.29 | 0.282 | 2 | 2.96 | 0.057 |
| *Residual SE (df)* | 0.22 (83) | | | 0.05 (89) | | | 0.15 (89) | | |
| 1. *CORT – life history at G42* | | | | | | | | | |
|  | *log(CORT)* | | | *log(developmental time)* | | | *log(mass)* | | |
| ***Factors*** | *Df* | *F* | *p* | *Df* | *F* | *P* | *Df* | *F* | *p* |
| *Population* | 2 | 2.57 | 0.082 | 2 | 9.00 | **<0.001** | 2 | 10.73 | **<0.001** |
| *pH* | 1 | 1.54 | 0.218 | 1 | 1.29 | 0.259 | 1 | 0.17 | 0.678 |
| *Block* | 1 | 2.10 | 0.151 | 1 | 14.67 | **<0.001** | 1 | 1.50 | 0.224 |
| *pop x pH* | 2 | 0.52 | 0.596 | 2 | 1.26 | 0.287 | 2 | 0.32 | 0.727 |
| *Residual SE (df)* | 0.32(84) | | | 0.06(88) | | | 0.13(88) | | |
| 1. *univariate ANOVAS from the morphology CORT – MANOVA* | | | | | | | | | |
| ***Factors*** | *log(body depth)* | | | *log(body length)* | | | *log(tail length)* | | |
| *Population* | 2 | 2.92 | 0.060 | 2 | 0.83 | 0.440 | 2 | 0.42 | 0.659 |
| *pH* | 1 | 0.95 | 0.334 | 1 | 0.667 | 0.417 | 1 | 8.28 | **0.005** |
| *Block* | 1 | 0.45 | 0.504 | 1 | 1.71 | 0.195 | 1 | 2.79 | 0.099 |
| *log(mass)* | 1 | 422.99 | **<0.001** | 1 | 368.60 | **<0.001** | 1 | 21.70 | **<0.001** |
| *pop x pH* | 2 | 0.07 | 0.936 | 2 | 0.45 | 0.638 | 2 | 0.39 | 0.676 |
| *Residual SE (df)* | 0.04(80) | | | 0.03(80) | | | 0.07(80) | | |
| ***Factors*** | *log(tail depth)* | | | *log(tail muscle depth)* | | | *log(CORT)* | | |
| *Population* | 2 | 6.60 | **0.002** | 2 | 7.34 | **0.001** | 2 | 11.98 | **<0.001** |
| *pH* | 1 | 0.38 | 0.542 | 1 | 8.87 | **0.003** | 1 | 2.67 | 0.106 |
| *Block* | 1 | 0.74 | 0.392 | 1 | 0.06 | 0.809 | 1 | 58.50 | **<0.001** |
| *log(mass)* | 1 | 176.27 | **<0.001** | 1 | 15.74 | **<0.001** | 1 | 3.30 | 0.073 |
| *pop x pH* | 2 | 1.79 | 0.174 | 2 | 0.98 | 0.381 | 2 | 0.33 | 0.073 |
| *Residual SE (df)* | 0.05(80) | | | 0.08(80) | | | 0.47(84) | | |


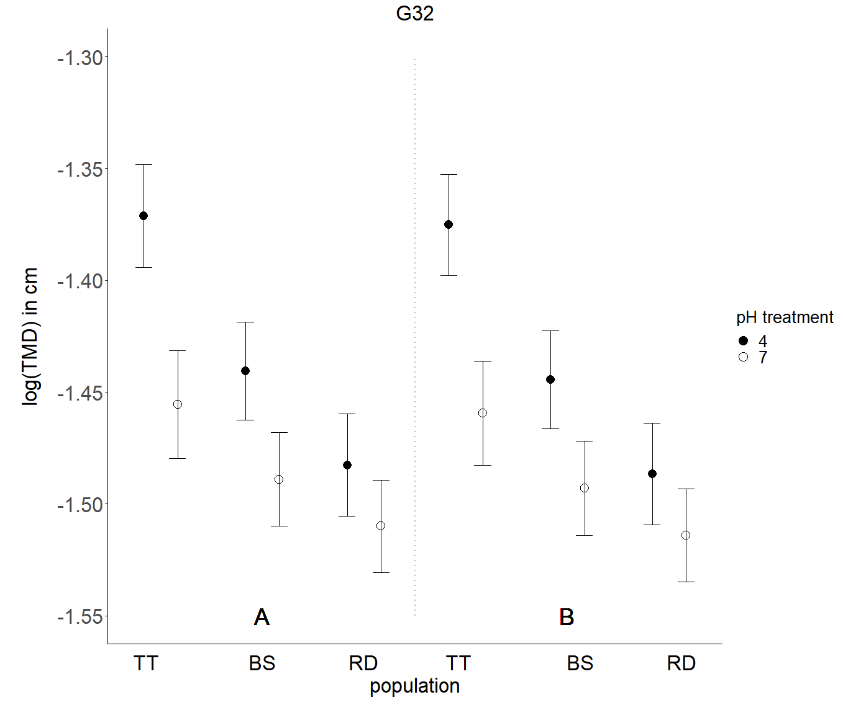

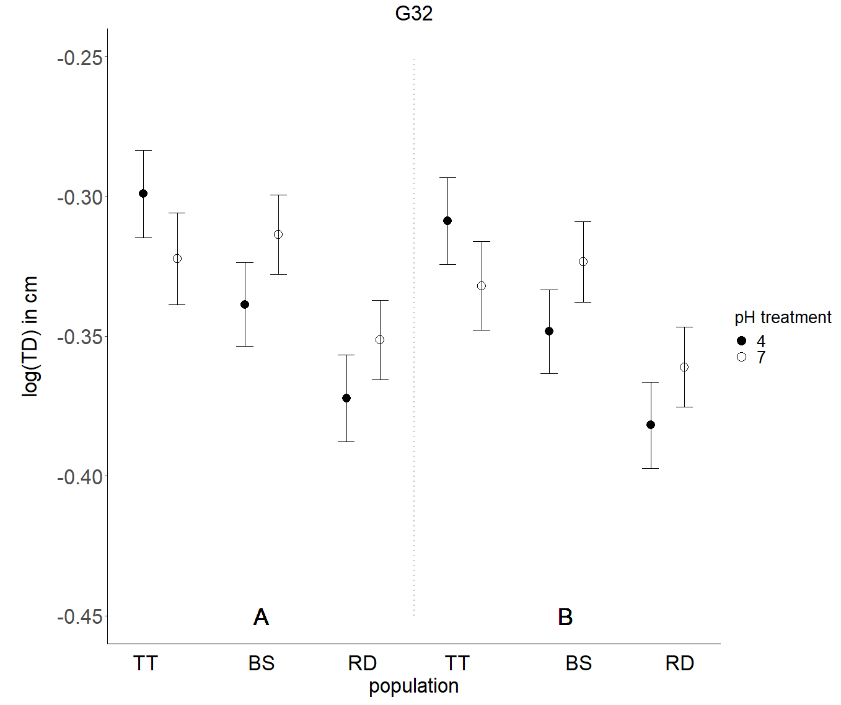

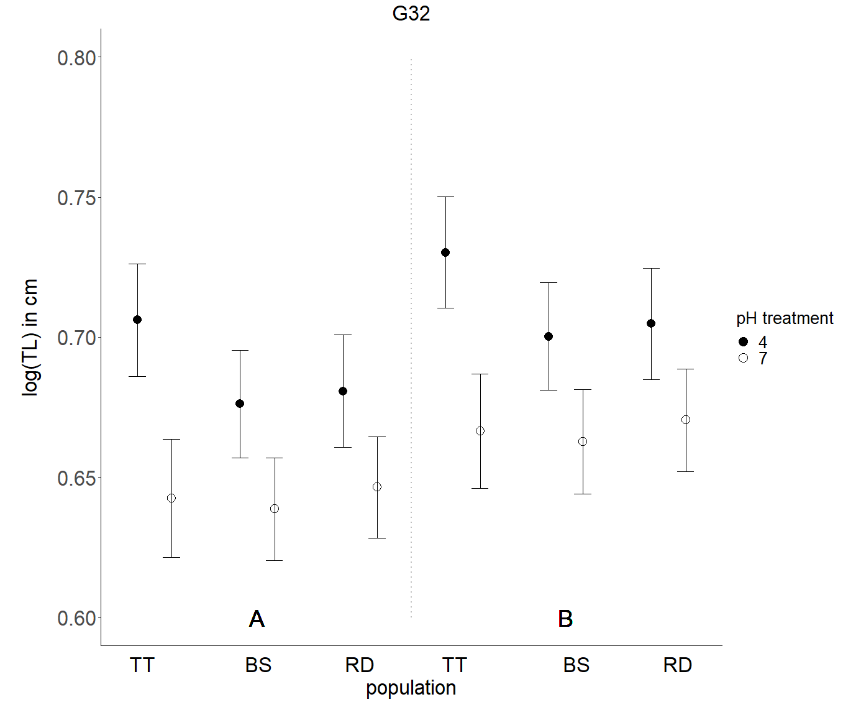

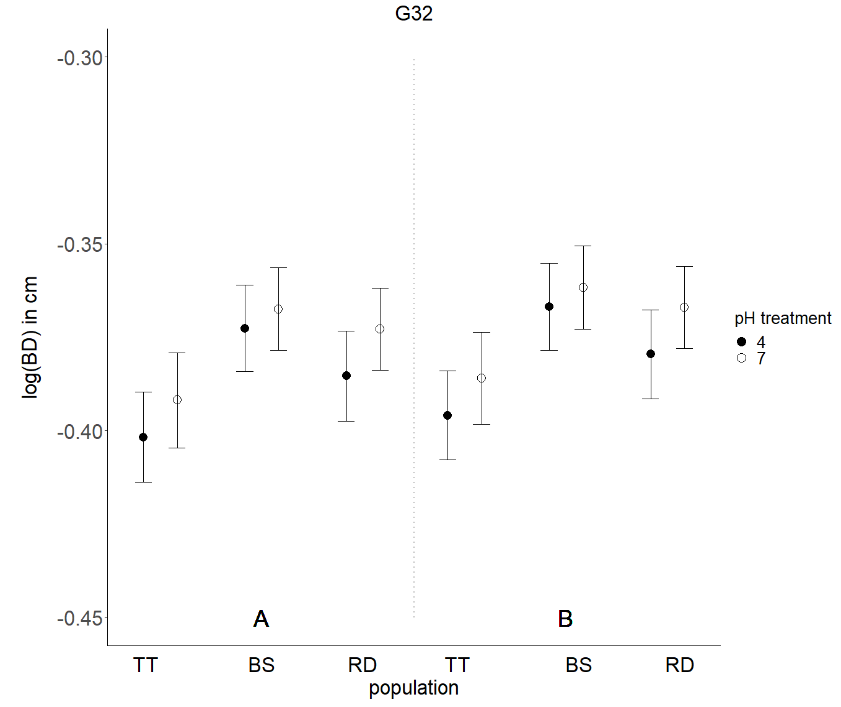

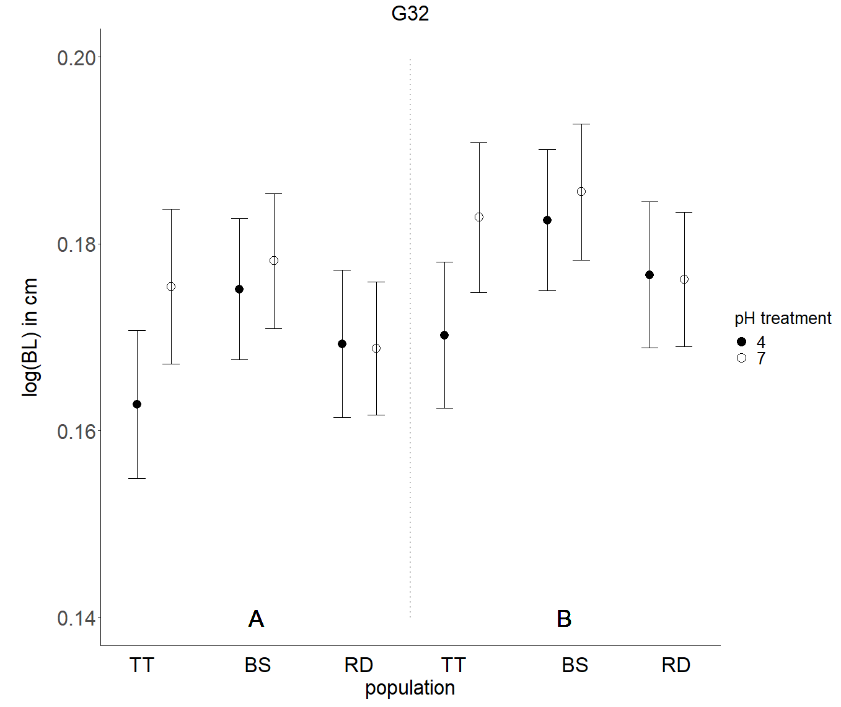
**Additional Figure 1.1.**

pH treatment

acid

neutral

a)

b)

c)

d)

e)

**Additional Figure 1.1:** LS mean ± SE of A) log(body length, BL), B) log(body depth, BD), C) log(tail length, TL), D) log(tail depth, TD) and E) log(tail muscle depth, TMD), corrected for tadpole mass at G32. The data are derived from the CORT-morphology MANOVA model (see main text and Additional Table 1.3 for details). Data is presented for three *Rana arvalis* populations (TT: acid, BS: intermediate, RD: neutral pH origin) reared in two pH treatments (acid: 4, neutral: 7) from G25 to G32. Block is indicated by x axis as: A = morning sampling/warmer temperature block, B = afternoon sampling/cooler temperature block.

These graphs show that tadpoles had generally longer bodies when reared in the B block (a) and relatively longer tails when reared in acidic pH (c). Moreover, TT tadpoles had relatively longer and deeper tails, deeper tail muscles and shallower bodies than the RD tadpoles. Tail morphology divergence was especially clear under acidic conditions. Interestingly, TT tadpoles had deeper tail muscles than RD tadpoles, and BS were intermediate, particularly when reared in acidic conditions (gradient pattern in e). Trait divergence between the TT and RD tadpoles was relatively consistent in both acid and neutral treatments, whereas for BS tadpoles trait values were less consistent between the two pH treatments in body and tail depth (BD and TD). For the univariate ANOVA results, see Additional Table 1.4. N = 8 except for RD4B, TT4B, TT7B (N= 7), TT4A (N = 6) and TT7A (N = 5).
