# Supplementary material for: Context dependent variation in corticosterone and phenotypic divergence of Rana arvalis populations along an acidification gradient
Source: BMC Ecol Evol. 2022 Feb 5;22:11. doi: 10.1186/s12862-022-01967-1 (PMC8818180; doi:10.1186/s12862-022-01967-1)
Supplement: Supplementary file 2 — Additional file 2: Canonical models and additional HE plots of MAN(C)OVAs [file 12862_2022_1967_MOESM2_ESM.docx]

**Additional file 2**

**Additional file 2: Canonical models and additional HE plots of MAN(C)OVAs at G32**

**Additional Table 2.1:** Canonical models of the CORT - Life history MANOVA

**Additional Table 2.2:** Canonical models of the CORT - Morphology MAN(C)OVA

**Additional Figure 2.1.** Canonical HE plots from life history A) and B) morphology MANCOVAs at G32.

**Additional Figure 2.2 a-j.** HE plots from MANOVA for pairwise morphological associations from CORT- morphology MANOVA at G32

**Additional file 2: Canonical models and additional HE plots of MAN(C)OVAs**

**Additional Table 2.1:** Canonical models from CORT - life history MANOVAs at G32 including the fixed factors Population, pH, Block and Population x pH interaction. These were run to examine the multivariate differences of *Rana arvalis* tadpoles across CORT, developmental time (dev.time) and mass. For each factor of the MANOVAs (see main text) the following was run: 1) a canonical discriminant analysis (*Can.disc.a.)*, 2) test of H0 (whether all canonical correlations in each row are zero), 3) Coefficients for each variable in the MANOVA of the Canonical discriminant component. Significant effects (p<0.05) are shown in bold. *Can R^2^* = proportion of variance of one variable that explains the variance in the second variable. Statistics were run in the R package candisc (main manuscript[101]).

| Population | | | | | |
| --- | --- | --- | --- | --- | --- |
| *Can.disc.a.* | *Can R ^2^* | *Eigenvalue* | *Difference* | *Percent of variation explained* | *Cumulative percent of variation explained* |
| *Can1* | 0.275 | 0.379 | 0.293 | 81.42 | 81.42 |
| *Can2* | 0.080 | 0.086 | 0.293 | 18.58 | 100 |
| *H0* | *LR test* | *approx F* | *num df* | *den df* | *P* |
| *Can1* | 0.667 | 6.50 | 6 | 174 | **<0.001** |
| *Can2* | 0.920 | - | 2 | - | **-** |
| *coefficient* | *Can1* | *Can2* | - | - |  |
| log(CORT) | -0.756 | -0.315 | - | - |  |
| log(dev.time) | 0.514 | -0.869 | - | - |  |
| log(mass) | 0.637 | 0.423 | - | - |  |
| pH | | | | | |
| *Can.disc.a.* | *Can R ^2^* | *Eigenvalue* | *Difference* | *Percent* | *Cumulative* |
| *Can1* | 0.211 | 0.268 | - | 100 | 100 |
| *H0* | *LR test* | *approx F* | *num DF* | *den DF* | *P* |
| *Can1* | 0.789 | 7.86 | 3 | 88 | **<0.001** |
| *coefficient* | *Can1* | - | - | - |  |
| log(CORT) | -0.288 | - | - | - |  |
| log(dev time) | -0.674 | - | - | - |  |
| log(mass) | 0.754 | - | - | - |  |
| Block | | | | | |
| *Can.disc.a.* | *Can R ^2^* | *Eigenvalue* | *Difference* | *Percent* | *Cumulative* |
| *Can1* | 0.542 | 1.181 | - | 100 | 100 |
| *H0* | *LR test* | *approx F* | *num DF* | *den DF* | *P* |
| *Can1* | 0.458 | 34.65 | 3 | 88 | **<0.001** |
| *coefficient* | *Can1* | - | - | - |  |
| log(CORT) | -0.865 | - | - | - |  |
| log(dev time) | 0.624 | - | - | - |  |
| log(mass) | 0.254 | - | - | - |  |
| population x pH | | | | | |
| *Can.disc.a.* | *Can R ^2^* | *Eigenvalue* | *Difference* | *Percent* | *Cumulative* |
| *Can1* | 0.020 | 0.020 | 0.011 | 68.04 | 68.04 |
| *Can2* | 0.009 | 0.009 | 0.011 | 31.96 | 100 |
| *H0* | *LR test* | *approx F* | *num DF* | *den DF* | *P* |
| *Can1* | 0.971 | 0.42 | 6 | 174 | 0.823 |
| *Can2* | 0.991 | - | 2 | - | - |
| *Coefficient* | *Can1* | *Can2* | - | - |  |
| log(CORT) | 0.146 | 0.901 | - | - |  |
| log(dev time) | -0.837 | 262 | - | - |  |
| log(mass) | -0.527 | -0.505 | - | - |  |

**Additional Table 2.2:** Canonical models of the CORT - Morphology MAN(C)OVA at G32 including fixed factors Population, pH, Block and Population x pH as well as covariate log(mass) and population x log(mass) interaction (see Table 3 in main text). These were run to examine multivariate differences of *Rana arvalis* tadpoles in CORT, body depth, body length, tail depth and tail muscle depth. For each factor of the MANOVAs (see main text), the following was run: 1) a canonical discriminant analysis (*Can.disc.a.)*, 2) test of H0 if all canonical correlations in each row are zero, 3) Coefficients for each variable in the MANOVA of the Canonical discriminant analysis. Significant effects (p<0.05) are shown in bold. *Can R^2^* = proportion of variance of one variable explains the variance of the second variable. Statistics were run in the R package candisc (main manuscript[101]).

| Population | | | | | |
| --- | --- | --- | --- | --- | --- |
| *Can.disc.a.* | *Can R ^2^* | *Eigenvalue* | *Difference* | *Percent* | *Cumulative* |
| *Can1* | 0.430 | 0.753 | 0.607 | 83.73 | 83.73 |
| *Can2* | 0.128 | 0.146 | 0.607 | 16.27 | 100 |
| *H0* | *LR test* | *approx F* | *num df* | *den df* | *P* |
| *Can1* | 0.498 | 5.50 | 12 | 158 | **<0.001** |
| *Can2* | 0.872 | 2.34 | 5 | 80 | **0.049** |
| *Coefficient* | *Can1* | *Can2* | - | - |  |
| *log(body depth)* | -0.396 | -0.660 | - | - |  |
| *log(body length)* | -0.317 | -0.758 | - | - |  |
| *log(tail length)* | 0.095 | 0.742 | - | - |  |
| *log(tail depth)* | 0.335 | -0.348 | - | - |  |
| *log(tail muscle depth)* | 0.451 | -0.585 | - | - |  |
| *log(CORT)* | -0.849 | -0.239 | - | - |  |
| pH | | | | | |
| *Can.disc.a.* | *Can R ^2^* | *Eigenvalue* | *Difference* | *Percent* | *Cumulative* |
| *Can 1* | 0.210 | 0.265 | - | 100 | 100 |
| *H0* | *LR test* | *approx F* | *num DF* | *den DF* | *p* |
| *Can 1* | 0.790 | 3.536 | 6 | 80 | **0.004** |
| *Coefficient* | Can1 | - | - | - |  |
| *log(body depth)* | 0.209 | - | - | - |  |
| *log(body length)* | -0.697 | - | - | - |  |
| *log(tail length)* | 0.794 | - | - | - |  |
| *log(tail depth)* | -0.242 | - | - | - |  |
| *log(tail muscle depth)* | 0.529 | - | - | - |  |
| *log(CORT)* | 0.158 | - | - | - |  |
| Block | | | | | |
| *Can.disc.a.* | *Can R ^2^* | *Eigenvalue* | *Difference* | *Percent* | *Cumulative* |
| *Can 1* | 0.450 | 0.820 | - | 100 | 100 |
| *H0* | *LR test stat* | *approx F* | *num DF* | *den DF* | *p* |
| *Can 1* | 0.550 | 10.93 | 6 | 80 | **<0.001** |
| *Coefficient* | Can1 | - | - | - |  |
| *log(body depth)* | -0.114 | - | - | - |  |
| *log(body length)* | -0.070 | - | - | - |  |
| *log(tail length)* | 0.446 | - | - | - |  |
| *log(tail depth)* | 0.138 | - | - | - |  |
| *log(tail muscle depth)* | -0.015 | - | - | - |  |
| *log(CORT)* | -1.027 | - | - | - |  |
| log(mass) | | | | | |
| *Can.disc.a.* | *Can R ^2^* | *Eigenvalue* | *Difference* | *Percent* | *Cumulative* |
| *Can 1* | 0.943 | 16.534 | - | 100 | 100 |
| *H0* | *LR test* | *approx F* | *num DF* | *den DF* | *p* |
| *Can 1* | 0.057 | 220.46 | 6 | 80 | **<0.001** |
| *Coefficient* | Can1 | - | - | - |  |
| *log(body depth)* | 0.929 | - | - | - |  |
| *log(body length)* | 0.802 | - | - | - |  |
| *log(tail length)* | -0.307 | - | - | - |  |
| *log(tail depth)* | 0.112 | - | - | - |  |
| *log(tail muscle depth)* | 0.398 | - | - | - |  |
| *log(CORT)* | 0.300 | - | - | - |  |
| population x pH | | | | | |
| *Can.disc.a.* | *Can R ^2^* | *Eigenvalue* | *Difference* | *Percent* | *Cumulative* |
| *Can 1* | 0.096 | 0.107 | 0.085 | 82.88 | 82.88 |
| *Can 2* | 0.022 | 0.022 | 0.085 | 17.12 | 100 |
| *H0* | *LR test stat* | *approx F* | *num DF* | *den DF* | *p* |
| *Can 1* | 0.884 | 0.837 | 12 | 158 | 0.613 |
| *Can 2* | 0.978 | 0.353 | 5 | 80 | 0.879 |
| *Coeffient* | Can1 | Can2 | - | - |  |
| *log(body depth)* | -0 | 0.445 | - | - |  |
| *log(body length)* | -0.842 | 0.119 | - | - |  |
| *log(tail length)* | 0.697 | -0.239 | - | - |  |
| *log(tail depth)* | 0.552 | -0.693 | - | - |  |
| *log(tail muscle depth)* | 0.298 | 0.774 | - | - |  |
| *log(CORT)* | -0.366 | -0.671 | - | - |  |

**Additional Figure 2.1**

**A)**


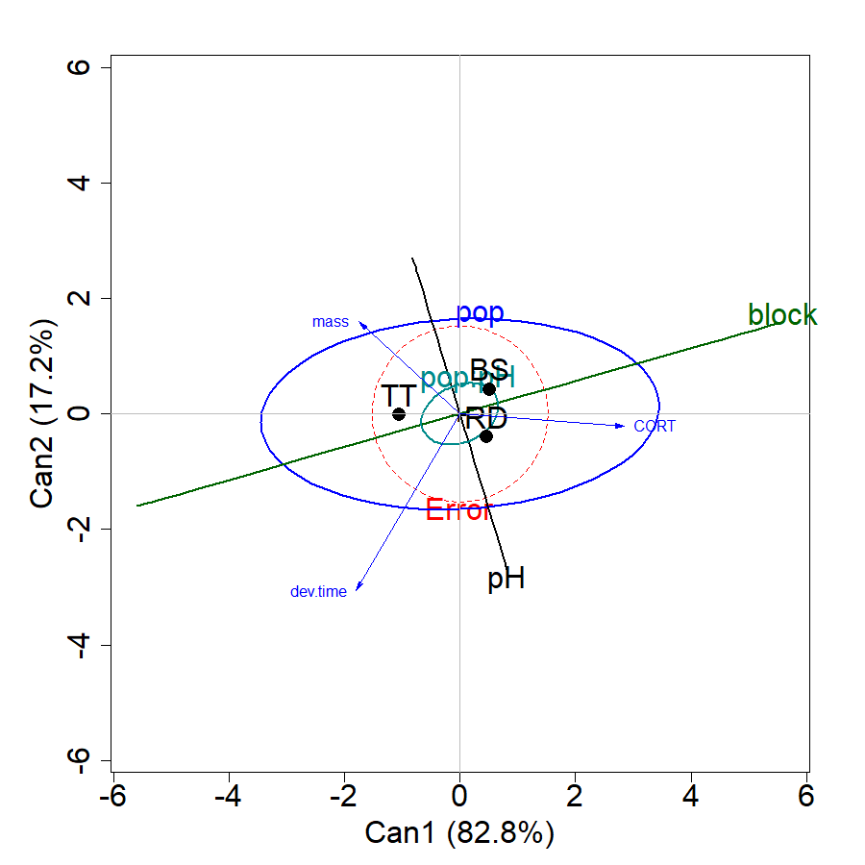


**B)**


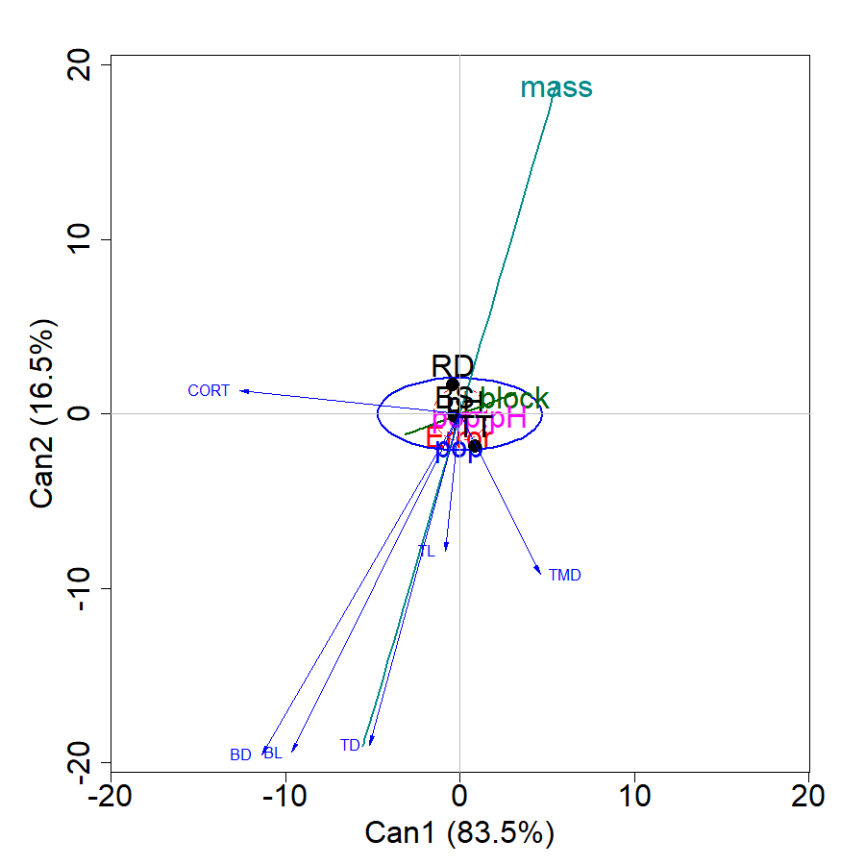


**Additional Figure 2.1** Canonical HE plots of A) life history MANOVA and B) morphology MANCOVA at mid larval stage G32. Hypothesis ellipsoids that are outside of the error (red) ellipse reflect significant effects. (See Additional file Tables 2.1. & 2.2. for details of models). Solid circles indicate Population means (TT: acid pH origin, BS: intermediate pH origin, RD: neutral pH origin). The blue arrows indicate the direction of different traits in the models. Fixed factors included Population (pop), Block (block), pH treatment (ph) and Population-pH treatment interaction (pop:ph). Response variables included are CORT, mass and developmental time (dev. time) in A, and CORT, tail length (TL), body depth (BD), tail depth (TD), body length (BL), tail muscle depth (TMD) in B. In B, mass was included as covariate. All traits were log transformed. Statistics were run in the R package candisc (main manuscript[101]). The following can be concluded from the canonical HE plot for morphological traits and CORT (Figure B): TT tadpoles were generally relatively larger in morphological traits (Population differences align with body mass and morphological traits), particularly in tail length and tail muscle depth. However, CORT was not overall associated with body mass or morphology (i.e. CORT arrow points in a different direction than other tadpole traits in the canonical HE plot). TT tadpoles had the lowest CORT values, followed by BS and RDs tadpoles. Based on the ellipsoids, Block, population, pH and body mass had a significant effect in the multivariate space.


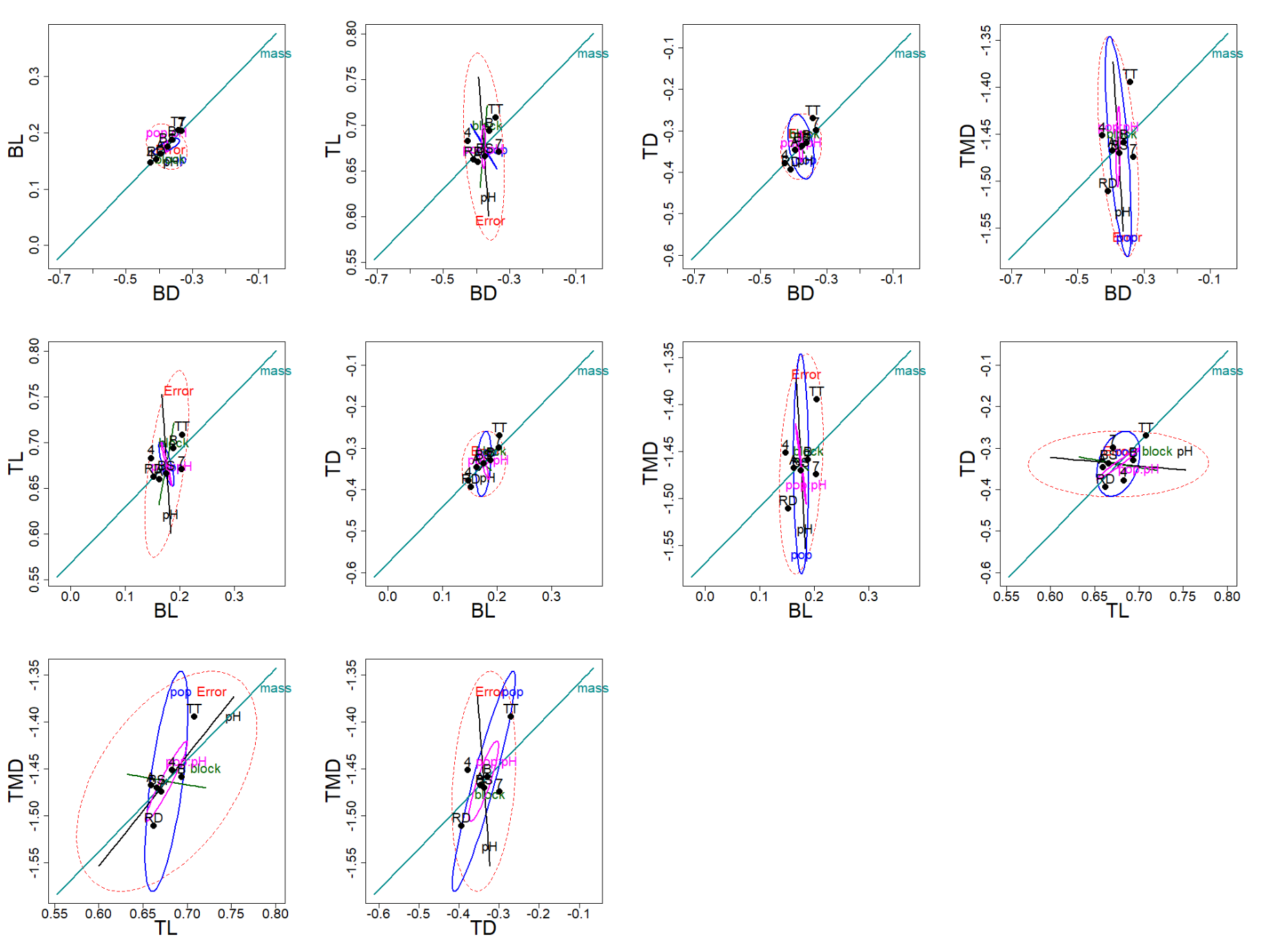


a)

b)

c)

d)

e)

f)

g)

h)

i)

j)

**Additional Figure 2.2 a-j** HE plots from MANOVA of CORT and tadpole morphology of *Rana arvalis* at G32 (see main text) for bivariate trait associations, with tadpole mass as covariate. (For CORT-trait associations see main text). All response variables were log transformed. Ellipsoids that are outside of the error ellipse reflect significant effects. Fixed effect ellipsoids for population (TT: acid pH origin, BS: intermediate pH origin, RD: neutral pH origin), pH treatment (4: acid and 7: neutral) and Block (A:morning sampling/warmer block), B: afternoon sampling/cooler block) are shown. Morphological traits included Body depth: BD, body length: BL, tail depth: TD, tail length: TL and tail muscle depth: TMD. All traits are strongly related to body mass. I) and j) also showed a significant effect of population mainly driven by tail muscle depth.
