# Supplementary material for: Context dependent variation in corticosterone and phenotypic divergence of Rana arvalis populations along an acidification gradient
Source: BMC Ecol Evol. 2022 Feb 5;22:11. doi: 10.1186/s12862-022-01967-1 (PMC8818180; doi:10.1186/s12862-022-01967-1)
Supplement: Supplementary file 3 — Additional file 3: Additional DAPC results [file 12862_2022_1967_MOESM3_ESM.docx]

**Additional file 3: Additional DAPC results**

**Additional Figure 3.1** Decision process for the DAPC models on CORT- morphology for A) G32 and G38 stages combined and B) only G32 stages.

**Additional Table 3.1** Contributions of CORT and tadpole morphology variable to the DAPC for A) G32 and G38 stages combined and B) only G32 stages.

**Additional Figure 3.2:** Overall DAPC graph and loading plots on CORT and morphology G32 and G38 stages.

**Additional Figure 3.1**

1.
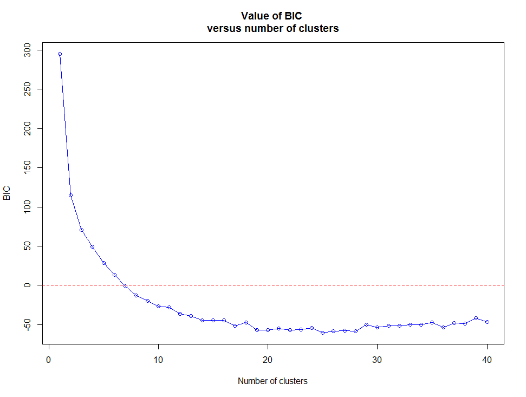

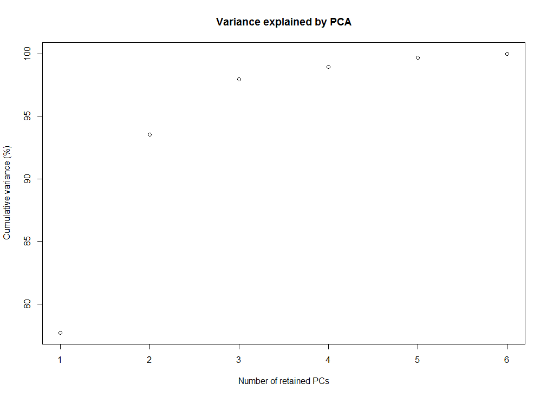

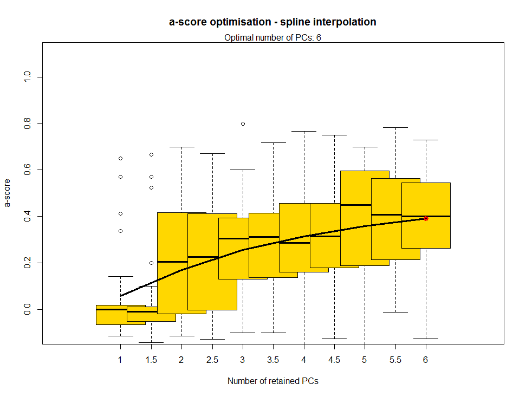
G32 & G38

1)
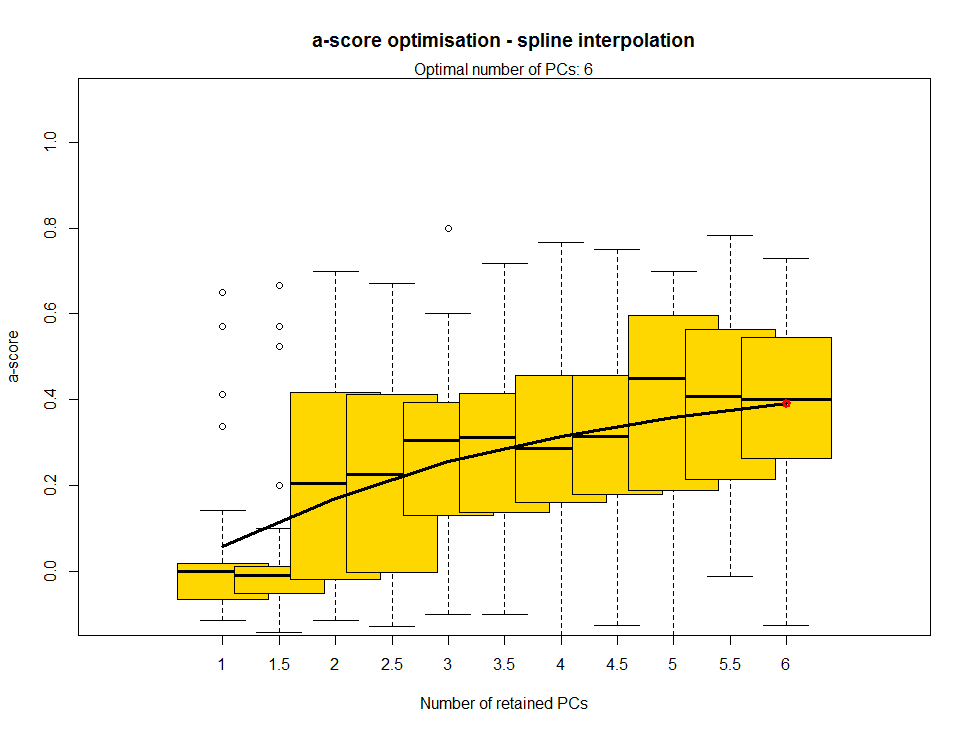


2)

3)

1.
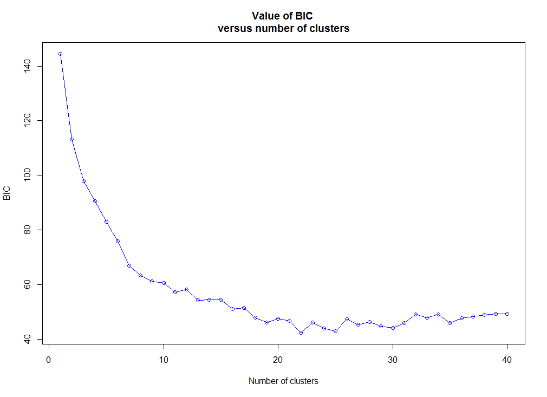

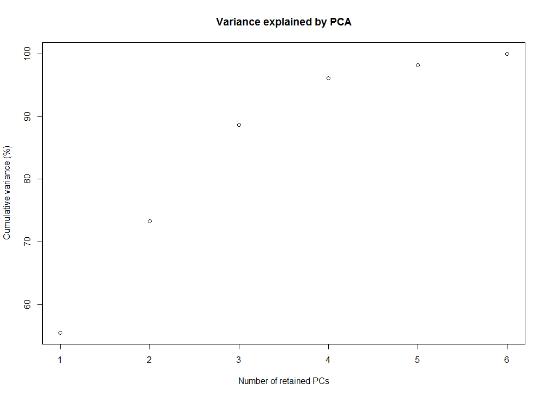

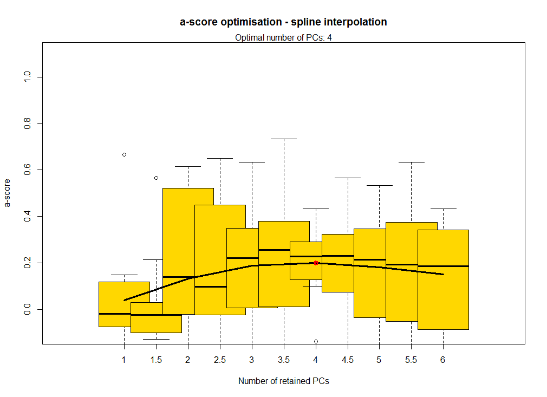
G32

1)

2)

3)

**Additional Figure 3.1** Decision process on how many principal components (PC) and discriminant axes (DA) for CORT and morphological traits should be retained for the DAPC models in A) combining G32 and G38 stages B) including only G32 stage. In each of 1) a-score optimisation curve (to avoid overfitting due to retaining too many PCs) 🡪 red dot indicates the number of PCs that should be considered, 2) variance explained by the PCs and 3) Bayesian Information Criterion (BIC) in relation to number of clusters 🡪 how many clusters should be retained. Plots were derived from the *adegenet* package in R (for further details see main text).

**Additional Table 3.1** Contributions of CORT and morphological variables to the DAPC for A) G32 & G38 stages combined and B) only G32 stage. Higher values stand for higher loading on the respective linear discriminants (LD1-LD6) (highest for relevant LDs are marked in bold). All traits were log transformed.

| 1. G32 & G38 | | | | | | |
| --- | --- | --- | --- | --- | --- | --- |
| Variable | LD1 | LD2 | LD3 | LD4 | LD5 | LD6 |
| log(body length) | **0.670** | **0.551** | 4.70e-01 | 0.226 | 0.375 | 6.10e-01 |
| log(body depth) | 0.019 | 0.001 | 9.21e-02 | 0.051 | 0.036 | 3.54e-01 |
| log(tail length) | 0.102 | 0.002 | 2.32e-01 | 0.211 | 0.225 | 2.58e-02 |
| log(tail muscle depth) | 0.198 | 0.009 | 9.42e-02 | 0.222 | 0.087 | 1.91e-03 |
| log(tail depth) | 0.010 | **0.358** | 2.03e-05 | 0.290 | 0.277 | 7.81e-03 |
| log(CORT) | 0.001 | 0.079 | 4.36e-04 | 0 | 0 | 5.22e-05 |
| 1. G32 | | | | | | |
| Variable | LD1 | LD2 | LD3 | LD4 |  |  |
| log(body length) | 0.011 | 0.008 | 0.048 | 0.096 |  |  |
| log(body depth) | 0 | **0.293** | 0.079 | 0.021 |  |  |
| log(tail length) | 0.037 | **0.379** | 0.270 | 0.846 |  |  |
| log(tail muscle depth) | **0.646** | 0.068 | 0.461 | 0.001 |  |  |
| log(tail depth) | **0.184** | 0.251 | 0.141 | 0.034 |  |  |
| log(CORT) | **0.122** | 0.001 | 0.001 | 0.003 |  |  |


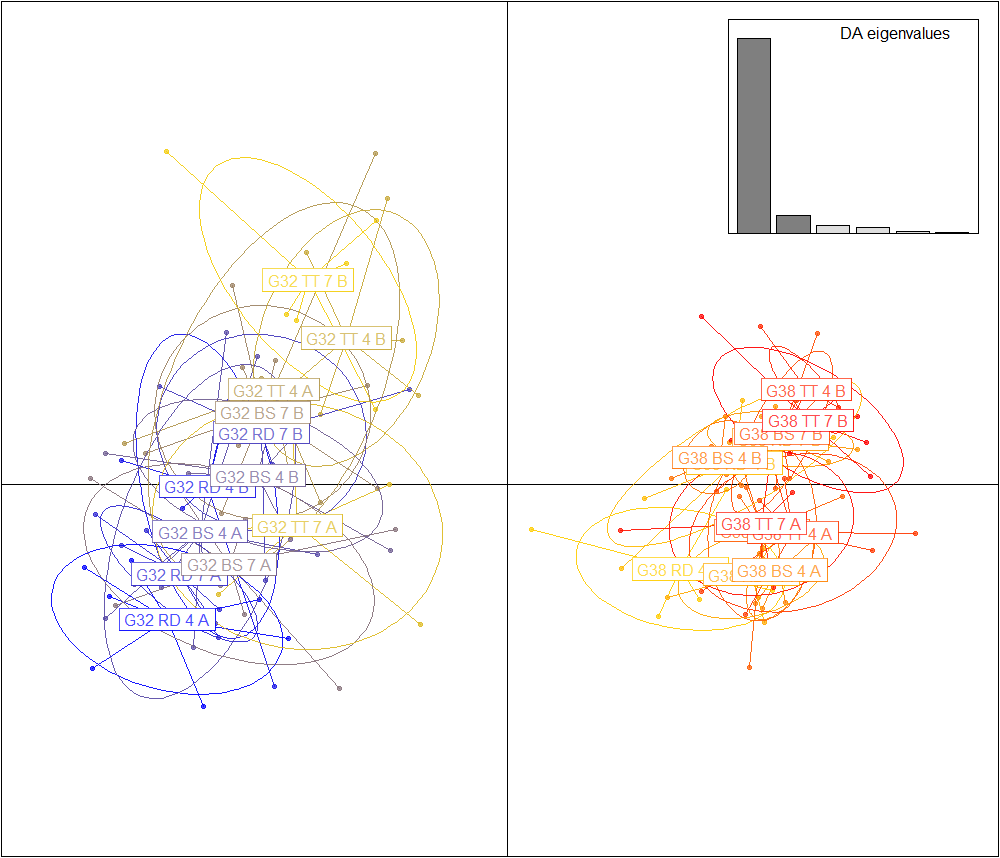


A)

C)

BL

BD

TL

TMD

TD

CORT

BL

BD

TL

TMD

TD

CORT

LD1

LD2


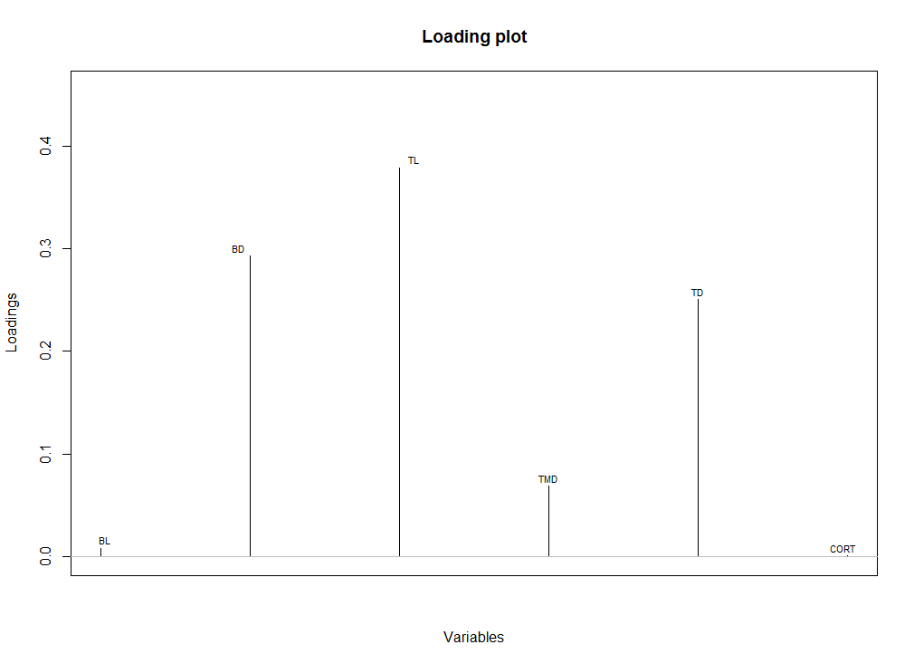

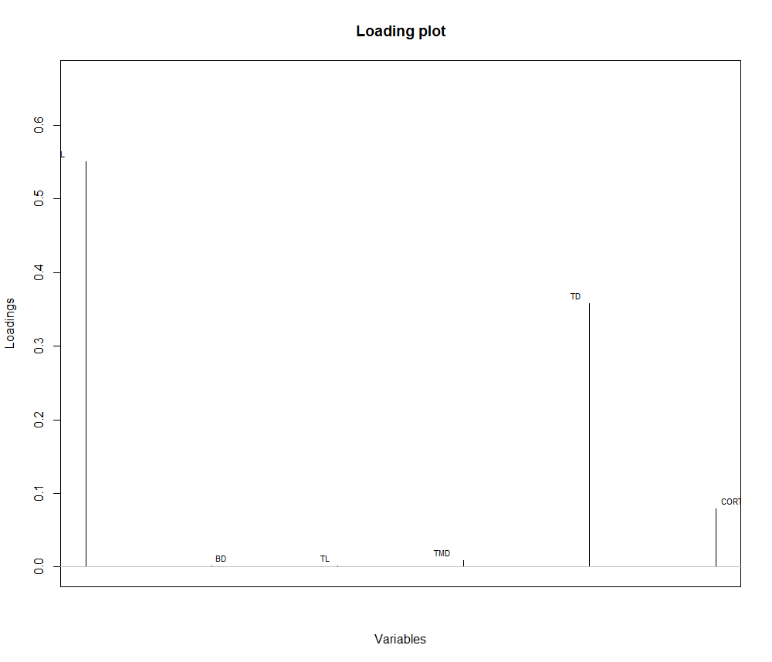


B)

**Additional Figure 3.2:** A) DAPC on overall morphology and respective loading plots for B) G32 and G38 stages and C) only G32 stage. The analysis included CORT and body length (BL), body depth (BD), tail length (TL), tail depth (TD) and tail muscle depth (TMD)) for *Rana arvalis* tadpoles at G32 and G38. Data included three populations (TT: acid pH origin, BS:intermediate pH origin, RD: neutral pH origin) reared in two pH treatments (‘4’ acid and ‘7’ neutral pH treatment) within two rearing blocks (A: morning sampling/warmer, B: afternoon sampling/colder). All traits were log transformed.

In A) LD1 (x-axis) explained 84.0% of the variance and represents mainly variation in body length, tail length and tail muscle depth, and LD2 (y-axis) explained 8.0 of the variance and represents mainly variation in tail depth and body length. See Additional Table 3.1 for further details. There was clearly more variation, particularly along the LD2 axis at G32, whereas at G38 observations were more aggregated, indicating more similar phenotypes. For combined G32 and G38 (B), tail depth, CORT and body length loaded strongest (threshold > 0.07). For G32 stage (C), body depth, tail muscle depth, tail length and tail depth loaded strongest (threshold above 0.07). For further details on G32 analyses, see main manuscript. N = 8 for each population- treatment combination, except for G32: RD4A, RD7A, RD7B and BS4B: N =7; RD4B, TT4B and TT7B: N = 6; TT4A and TT7A: N = 5; G38: RD4A, RD7A, RD7B, BS4A, BS7B, TT7A and TT7B: N =7; RD4B, BS4B and TT4B: N = 6Lower replicate number was due to mortality or missing trait value (See Methods for details).
