# Supplementary material for: Context dependent variation in corticosterone and phenotypic divergence of Rana arvalis populations along an acidification gradient
Source: BMC Ecol Evol. 2022 Feb 5;22:11. doi: 10.1186/s12862-022-01967-1 (PMC8818180; doi:10.1186/s12862-022-01967-1)
Supplement: Supplementary file 4 — Additional file 4: Validating the hormonal sampling and assay methods [file 12862_2022_1967_MOESM4_ESM.docx]

**Additional file 4 Validating the hormonal sampling and assay methods**

**Background**

When one starts hormonal analyses on a new species, appropriate sampling, extraction, and assay methods for glucocorticoid hormonal levels need to be established [1]. Corticosterone (CORT) measurements of tadpoles have so far been performed via heart-puncture (i.e. plasma collection as representation of short term hormonal state), sampling whole individuals (representing chronic hormonal state across all tissues) or sampling tail tissue (representing chronic hormonal state, but providing a less noisy sample than fully body samples which integrate all metabolic processes and tissue to use for specific questions related to tail expression) [e.g. 2, 3, 4]. The main hormone that has been used to measure stress responses in amphibians is corticosterone, which is the primary glucocorticoid produced in amphibians (reviewed in [1]). However, the relative proportion of corticosterone and cortisol, another glucocorticoid, can depend on developmental stage of tadpoles as well as the species [5]. As no prior studies on CORT levels in *Rana arvalis* have been published, to our knowledge, the following validation steps were used in our study: validation of relevant tissue, validation of primary glucocorticoid and validation of extraction method. As non-invasive sampling methods are not yet well established for tadpoles (but see [6] for a recent review), we validated invasive sampling methods for corticosterone/cortisol.

**Material and Methods**

For reliable assessment of hormonal variation, and determination of the primary glucocorticoid (i.e. corticosterone or cortisol) we conducted several small experiments, which were conducted under the same ethical and sampling permits as mentioned in the main text.

*1) Comparison of different tissues*

The goal of this experiment was to compare CORT assays of different tissue types (plasma, tail, body) and evaluate if they respond proportionally similarly to experimentally manipulated corticosterone levels (110uM) added for a subgroup of experimental animals via their housing water. This aided in deciding for the sampling technique (type of tissue/plasma in invasive sampling) to be used (Main manuscript).

In order to determine which type of sample to use, tadpoles were reared under same general experimental conditions as mentioned in Material and Methods of the main manuscript, except that rearing from egg collection onwards took place in a 19°C room (which induced faster development). Neutral reconstituted soft water (RSW) (pH 7.5, Henceforth: Neutral’7’) and acid RSW (pH 4.3, Henceforth: Acid’4’) was used as media from G25 stage onwards and prepared as described in the main text. We conducted a factorial experiment, where tadpoles were exposed at early to mid-larval stages (G29-G32) for 6 days to either “corticosterone water” (RSW with 110uM corticosterone (Sigma 27840) dissolved in 99.9% EtOH; henceforth: CORT), “vehicle control” water (RSW with EtOH (total 0.02% - equivalent to CORT treatment), Henceforth: vehicle control) or “control water” (untreated RSW, Henceforth: control). Water change (and therefore new exposure) was conducted every 2^nd^ day.

To account for biological variability within *R. arvalis*, we used individuals from four populations. Three of them were the same as in main manuscrip (TT: acid pH origin, BS: intermediate pH origin, RD: neutral pH origin). In addition we used SR (another previously studied neutral pH origin population, with breeding pond pH 7.3, Stubberud, see [7]). We exposed tadpoles from each of these populations to one of six treatments: Neutral.CORT; Neutral.vehicle control; Neutral.control; Acid.CORT; Acid.vehicle control; Acid.control. The individuals were chosen from a subset of 2-4 full-sib families/population. The following treatment/population level replications were set up: Neutral.CORT: 4 individuals/population, Neutral.vehicle control: 2 ind./pop., Neutral.control: 2 ind. /pop, Acid.CORT: 2 individuals/population, Acid.vehicle control: 1 ind./pop., Acid.control: 1 ind. /pop (Total N=48). Tadpoles were fed with spinach-spirulina mix in the same way as mentioned in the main manuscript. After the 6 day exposure, developmental stage of each individual was recorded visually (as in main manuscript), each individual was deeply anesthesized in 2g/L MS-222 (see main manuscript for the procedure), and the following samples taken [8, 9]: plasma (by heart-puncture) (which was followed by sacrificing of the animal by decapitation), tail tissue and body remains (i.e. without tail) following the methods of Gomez-Mestre [10] and Burraco [2]. CORT extraction and EIA was conducted the same way as mentioned in the main text. The mean duplicate sample coefficient of variation (CV%) was 2.85%.

*2) Biological validation and glucocorticoid assay validation for invasive sampling*

In order to test if the *activation* of the HPI axis [11] is detectable in the used assays (i.e. instead of representing noise and interactions of other metabolites), we validated that the hormonal values measured indeed represent the desired primary hormones. This was achieved by Liquid chromatography–mass spectrometry (LC-MS) (Add. Figure 1.3), a method that combines MS and HPLC. For LC-MS, the method description in Murtagh *et al.* [12] was followed. LC-MS was conducted by staff of the Endocrinology laboratory of Tobias Deschners’ laboratory, at the Max Planck Institute for Evolutionary Anthropology, Leipzig, Germany. After determining that CORT is the main glucocorticoid in our samples, we tested if this CORT, measured via LC-MS and extracted again from the respective columns of the LC-MS, was detectable in the EIA used (Arbor assay Cortictosterone DetectX, see main mauscript).

**Results & Conclusion**

*1) Comparison of different tissues*

CORT levels across all individuals in different tissues were correlated with each other (r > 0.63 in all pairwise correlations; Add Fig. 4.1) and all tissues also showed elevated levels in the CORT treatments (Acid.CORT, Neutral.CORT). This indicates that different tissues gave comparable results, although they are expected to reflect different time windows for CORT expression. Plasma levels, if sampled quickly, should reflect current baseline levels and, as all CORT samples derived from blood samples, should be well-suited for measuring short-term responses to stressors. Whole body samples can be sampled very quickly and should reflect long term hormonal responses (see [e.g. 2, 13, 14, 15] for general sampling comparisons). It is to be noted that in body samples plasma CORT levels are diluted and include also other cellular hormones. Our results show that CORT manipulation was effective and demonstrable in all tissues in the time frame of six days of exposure to CORT.

CORT levels in plasma were highest per unit volume (Add. Fig. 4.2). Plasma collection is logistically challenging in relatively small species, such as *R. arvalis* (tadpole mass post-hatching ca. 0.1-0.6 g), and took about 5 minutes (sampling/handling time of more than 3 minutes can affect CORT levels using this method, [e.g. 16, 17]). Values from tail tissue were sometimes below detection limits in the EIA (see main manuscript for characteristics of assay used) and also showed a massive range for the CORT treated individuals (Add Fig. 4.2). In contrast, body samples were in the detectable range most of the time and showed less within treatment variability in CORT levels (Add Fig. 4.2). Hence, as body samples are easy to obtain, show detectable responses to CORT manipulation, represent an integrative tissue for chronic CORT level monitoring and could be sampled fast, we used whole body sampling for further validation (See below) and in the main experiment (Main text).

a)

*r*= 0.68, *P*<0.010

b)

*r*= 0.73, *P*<0.010

*r*= 0.63, *P*<0.010

c)

**Additional Figure 4.1** Regression plots for CORT levels (pg/ml) in different tissues originating from the same individual *R. arvalis* tadpole: a) body (i.e. tissue without tail) against plasma, b) body against tail tissue and, c) plasma against tail tissue. The Pearson correlation coefficient (*r*) and *p* are indicated across all sampled tadpoles.


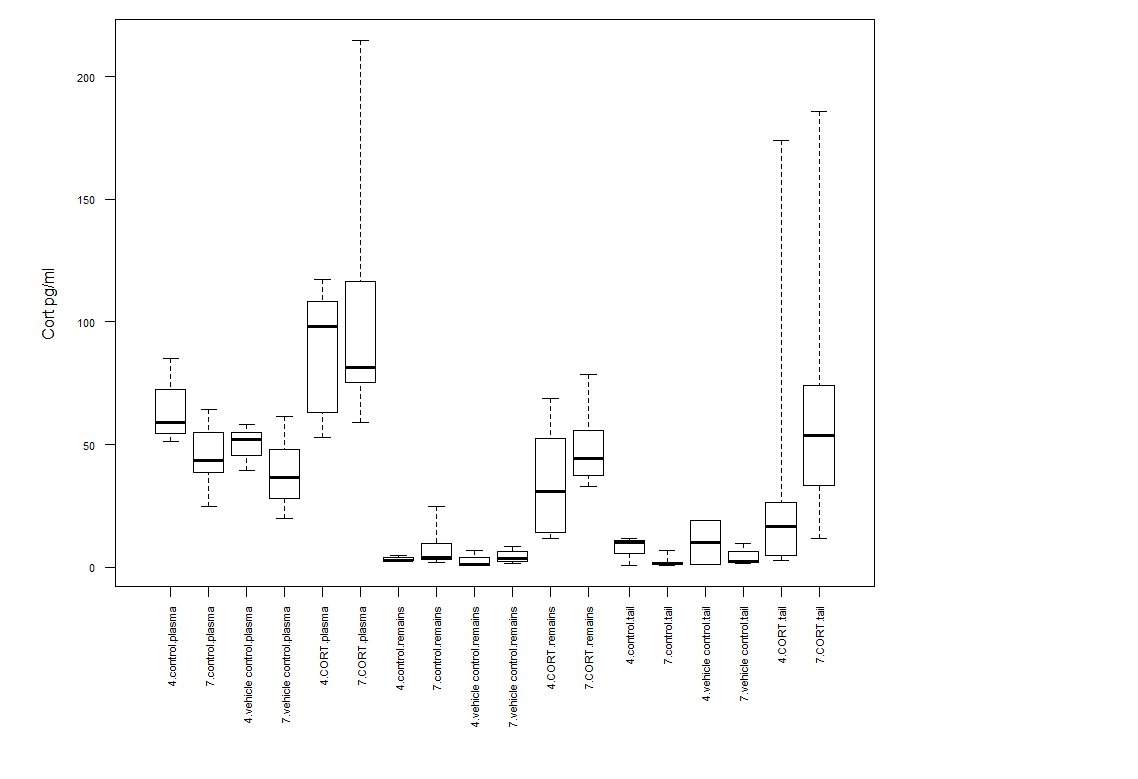


**Additional Figure 4.2** Box plot visualizing the CORT levels (pg/ml) of different tissues (plasma, body, tail). Each tissue was sampled for a given individual across 32 individuals in the following treatment group combinations: acid pH treatment (4) or neutral pH treatment (7), control, vehicle control and CORT treatment (110uM dissolved in EtOH). The median of data (horizontal line) is presented. 50% of the data distribution lies within the box and the whiskers represent the minimum and maximum of CORT levels measured.

*2) Biological validation and glucocorticoid assay validation*

CORT was the dominant glucocorticoid extracted from the six whole body samples of *R. arvalis* tadpoles (Add. Fig. 4.3). Samples extracted from all columns of the LC-MS were also measured using the CORT EIA (Arbor assay, see main text). Two representative examples (Add. Fig. 4.3) which had detectable levels of CORT in the LC-MS, also had detectable levels in the Corticosterone EIA analyses (levels estimated to be 680 pg/ml and 536 pg/ml- not corrected for tadpole body mass). The remaining four samples, also showed peaks for CORT in the LC-MS, but those were less clear, more noisy and partially below detectable limits compared to the other two samples. Those samples also were at the detection limits of the EIA. These results indicate that CORT was a primary, quantifiable glucocorticoid in *R. arvalis* tadpoles and that Corticosterone EIAs is an appropriate tool to measure glucocorticoid levels of this species, as no other convincing glucocorticoid was determined by LC-MS. Studies on other *Rana* species have shown that injected ACTH, a hormone involved in the Corticosterone/Cortisol synthesis and secretion, can activate the HPI axis (increase in CORT levels) in tadpoles (e.g. *Rana pipiens*, [e.g. 18]).


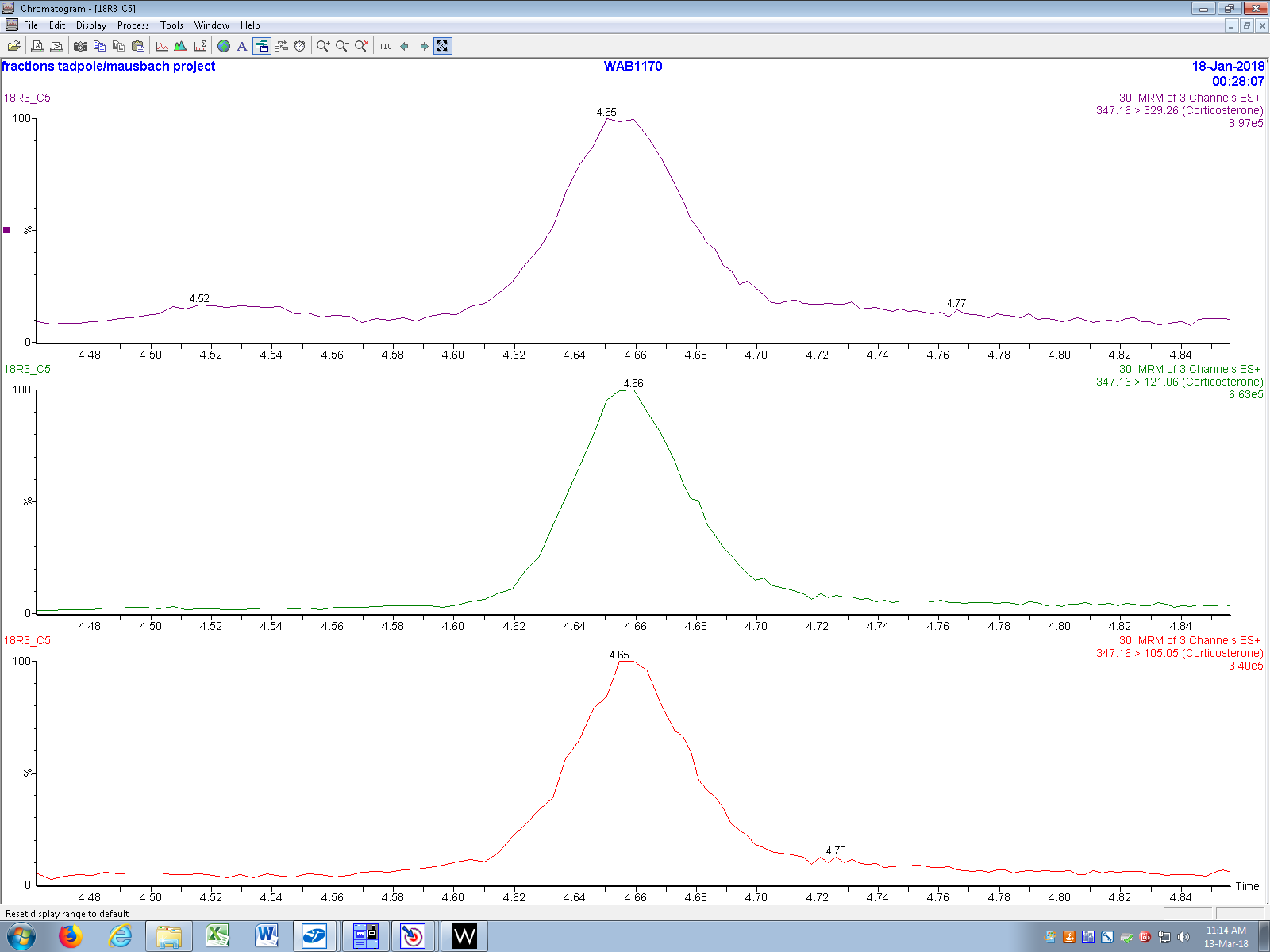


sample 1166


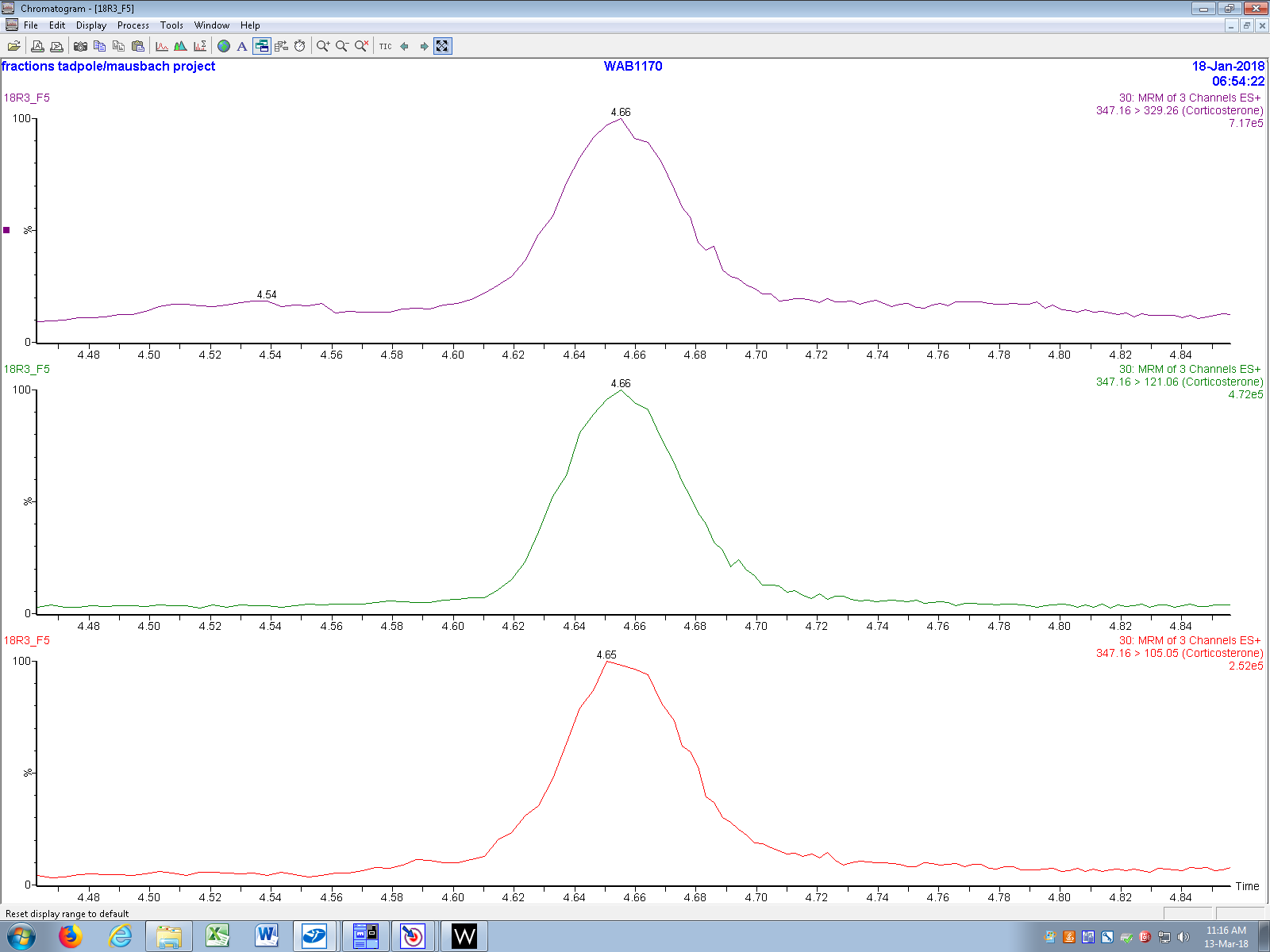


sample 1172

**Additional Figure 4.3** Two representative samples of glucocorticoids measured via LC-MS from whole body samples of *R. arvalis* tadpoles. The mass spectrometer searched for the three most abundant ions of corticosterone within each fraction (in this case fraction 5), where most corticosterone was found. The LC-MS analyses were done at the Endocrinology Laboratory of Max Planck Institute for Evolutionary Anthropology by Roisin Murtagh.

**Acknowledgements**

We especially thank Ivan Gomez-Mestres’ laboratory for teaching us the heart puncture method and the support throughout the validation. A tremendous thank goes to Roisin Murthagh and Tobias Deschner, conducting the LC-MS of our samples. Thanks also to Wolfgang Goymann recommending validation methods for our study.

**References**

1. Narayan, EJ. Non-invasive reproductive and stress endocrinology in amphibian conservation physiology. Conserv Physiol. 2013;1.
2. Burraco P, Arribas R, Kulkarni SS, Buchholz DR, Gomez-Mestre I. Comparing techniques for measuring corticosterone in tadpoles. Curr Zool. 2015;61: 835-45.
3. Glennemeier KA, Denver RJ. Small changes in whole-body corticosterone content affect larval Rana pipiens fitness components. Gen Comp Endocrinol. 2002a;127:16-25.
4. Hill R, Wyse G, Anderson M. Animal Physiology. Sunderland, MA,USA: Sinauer Associate.2008
5. Jungreis AM, Huibregtse WH, Ungar F. Corticosteroid identification and corticosterone concentration in serum of Rana pipiens during dehydration in winter and summer. Comp Biochem Physiol. 1970;34:683-9.
6. Narayan, E., Forsburg, Z., Davis, D., Gabor, C. (2019). Non-invasive methods for measuring and monitoring stress physiology in imperiled amphibians. Frontiers in Ecology and Evolution. Behavioural and Evolutionary Ecology. Front Ecol Evol. 2019;7:431.
7. Hangartner S, Laurila A, Räsänen K. Adaptive divergence in moor frog (Rana arvalis) populations along an acidification gradient: inferences from QST–FST correlations. Evolution. 2012;66: 867-81.
8. Ramlochansingh C, Branoner F, Chagnaud BP, Straka H. Efficacy of tricaine methanesulfonate (MS-222) as an anesthetic agent for blocking sensory-motor responses in Xenopus laevis tadpoles. PLoS One. 2014;9:e101606.
9. Cakir Y, Strauch SM. Tricaine (MS-222) is a safe anesthetic compound compared to benzocaine and pentobarbital to induce anesthesia in leopard frogs (Rana pipiens). Pharmacol Rep. 2005;57:467-74.
10. Gomez-Mestre I, Kulkarni S, Buchhol DR. Mechanisms and consequences of developmental acceleration in tadpoles responding to pond drying. 2013;PLoS One 8:e84266.
11. Denver, RJ. Stress hormones mediate environment-genotype interactions during amphibian development. Gen Comp Endocrinol. 2009;164:20-31.
12. Murtagh R, Behringer V, Deschner T. LC-MS as a method for non-invasive measure-ment of steroid hormones and their metabolites in urine and faeces of animals. Wien Tierärztl Monat – Vet Med Austria.2013;100:247–54.
13. Goymann W. Noninvasive monitoring of hormones in bird droppings: physiological validation, sampling, extraction, sex differences, and the influence of diet on hormone metabolite levels. Ann N Y Acad Sci. 2005;1046:35-53.
14. Nemeth M, Pschernig E, Wallner B, Millesi E. Non-invasive cortisol measurements as indicators of physiological stress responses in guinea pigs. PeerJ. 2016;4:e1590.
15. Touma C, Palme R. Measuring fecal glucocorticoid metabolites in mammals and birds: the importance of validation. Ann N Y Acad Sci. 2005;1046:54-74.
16. Cash WB, Holberton RL, Knight SS. Corticosterone secretion in response to capture and handling in free-living red- eared slider turtles. Gen Comp Endocrinol. 1997;108:427-33.
17. Romero LM, Romero RC. Corticosterone responses in wild birds: The importance of rapid initial sampling. The Condor. 2002;104:129-35
18. Glennemeier KA, Denver RJ. Developmental changes in interrenal responsiveness in anuran amphibians. Integr Comp Biol. 2002b;42:565-73.
